# Identification and Immunogenicity of African Swine Fever Virus Antigens

**DOI:** 10.3389/fimmu.2019.01318

**Published:** 2019-06-19

**Authors:** Christopher L. Netherton, Lynnette C. Goatley, Ana Luisa Reis, Raquel Portugal, Rachel H. Nash, Sophie B. Morgan, Lynden Gault, Raquel Nieto, Veronica Norlin, Carmina Gallardo, Chak-Sum Ho, Pedro J. Sánchez-Cordón, Geraldine Taylor, Linda K. Dixon

**Affiliations:** ^1^The Pirbright Institute, Woking, United Kingdom; ^2^Gift of Life Michigan Histocompatibility Laboratory, Ann Arbor, MI, United States; ^3^European Union Reference Laboratory for ASF, Centro de Investigación en Sanidad Animal-Instituto Nacional de Investigación y Tecnología Agraria y Alimentaria, Madrid, Spain

**Keywords:** T-cell, antigen presentation, swine, African swine fever (virus), disease enhancement, ELISPOT assay for interferon gamma, viral replication

## Abstract

African swine fever (ASF) is a lethal haemorrhagic disease of domestic pigs for which there is no vaccine. Strains of the virus with reduced virulence can provide protection against related virulent strains of ASFV, but protection is not 100% and there are concerns about the safety profile of such viruses. However, they provide a useful tool for understanding the immune response to ASFV and previous studies using the low virulent isolate OUR T88/3 have shown that CD8+ cells are crucial for protection. In order to develop a vaccine that stimulates an effective anti-ASFV T-cell response we need to know which of the >150 viral proteins are recognized by the cellular immune response. Therefore, we used a gamma interferon ELIspot assay to screen for viral proteins recognized by lymphocytes from ASF-immune pigs using peptides corresponding to 133 proteins predicted to be encoded by OUR T88/3. Eighteen antigens that were recognized by ASFV-specific lymphocytes were then incorporated into adenovirus and MVA vectors, which were used in immunization and challenge experiments in pigs. We present a systematic characterization of the cellular immune response to this devastating disease and identify proteins capable of inducing ASFV-specific cellular and humoral immune responses in pigs. Pools of viral vectors expressing these genes did not protect animals from severe disease, but did reduce viremia in a proportion of pigs following ASFV challenge.

## Introduction

African swine fever (ASF) is a haemorrhagic disease of domestic swine that limits the development of the pig industry in countries within which the disease is endemic, particularly sub-Saharan Africa. The virus (ASFV) is a threat to the rest of the world as shown by the spread of the disease from Georgia, through Russia, Belarus, and Ukraine to Czechia, Estonia, Hungary, Latvia, Lithuania, Poland, and Romania. The disease has recently appeared in Belgium, Vietnam, and throughout China and so threatens the national herd of the largest pig producing country in the world. Due to the lack of an effective vaccine, options for control are limited to quarantine and slaughter of pigs on infected farms. Inactivated virus does not protect against ASFV challenge even when used in combination with modern adjuvants (1). Although live attenuated viruses can provide robust immunity against related viruses (2–7) concerns remain due to the potential occurrence of a chronic form of ASF (2, 8, 9).

A subunit vaccine against ASFV would alleviate safety concerns attached to the use of live attenuated viruses and the protective efficacy of different combinations of ASFV antigens have been tested using various delivery mechanisms. Baculovirus expressed p30, p72, p54, and p22 (encoded by the CP204L, B646L, E183L, and KP177R genes, respectively) induced neutralizing antibodies and delayed the occurrence of clinical signs after challenge with the Pretoriuskop 96/4 strain of ASFV, but ultimately did not protect the animals from severe disease (10). Immunization with the virally encoded CD2 homolog (CD2v/EP402R) expressed by baculovirus induced antibodies capable of inhibiting haemadsorption and resulted in some protection against challenge with virulent ASFV (11). DNA vaccination with a plasmid encoding a fusion of the extracellular domain of CD2v (EP402R) with p30 (CP204L) and p54 (E183L) induced partial protection against the normally lethal E75 isolate when the open reading frame was fused to ubiquitin (12). In another experiment a library of ASFV genomic DNA fragments, again fused to ubiquitin, also induced partial protection against challenge with virulent ASFV (13). The immunity induced by both of these DNA vaccination studies was linked to cellular immunity in the absence of an antibody response. The immunogenicity of 40 different ASFV genes were tested by immunizing pigs with pool of plasmids followed by boosting with pools of recombinant vaccinia viruses expressing 47 antigens derived from 40 different ASFV genes (14). A number of novel antigens were identified and immunized pigs subsequently challenged with virulent ASFV showed reduced viremia and viral load in some tissues. More recent work has demonstrated the immunogenicity of replication deficient adenovirus (rAd) and modified vaccinia Ankara (MVA) expressing a number of ASFV antigens, including p72 (B646L), p54 (E183L), p30 (CP204L) and pp62 (CP530R) (15–17).

An alternative approach to ASFV antigen discovery would be to screen pigs protected from lethal challenge with virulent isolates after immunization with low virulence strains of ASFV. The choice of an appropriate screen is complicated by our poor understanding of the mechanisms of protection. A robust antibody response to a number of viral proteins is induced in animals immunized with live attenuated viruses (18, 19) that is capable of neutralizing virus (20) and passive transfer of serum containing neutralizing antibodies can protect against challenge with virulent ASFV (21). However, neutralizing antibodies alone are not sufficient for protection (10), and so it is likely that other mechanisms are involved such as the induction of antibodies capable of antibody-dependent cellular cytotoxicity (22) or inhibiting haemadsorption (23, 24). The cellular immune response plays an important role in protection [reviewed in Takamatsu et al. (25)] and is highlighted by the observations that secretion of IFNγ by lymphocytes (4) in response to recall antigen is a correlate of protection in pigs immunized with the low virulence isolate OUR T88/3 and proliferation of CD8 T-cells in response to recall antigen is a correlate of protection induced by ASFV strains BA71 ΔCD2V and E75CV1 (6, 26). Although the IFNγ response does not correlate with the protection afforded by all attenuated strains of ASFV (5, 6, 27), depletion of CD8^+^ lymphocytes abrogates the protection afforded by OUR T88/3 (3), strongly implicating a role for the cellular immune response in protection. P30 (CP204L), p72 (B646L) and the protein product of the G1340L gene are recognized by cytotoxic T cells (28–30) and I329L can induce proliferation of CD8+ lymphocytes (31). It is likely that pigs recovered from ASFV recognize additional proteins and that these could be a source of protective antigens for incorporation into a subunit vaccine. Screening overlapping peptide libraries represents a simple and unbiased approach to identify both MHC class I and class II restricted antigens (32). Due to the correlation between protection in the OUR T88/3 model and the IFNγ response to recall antigen we elected to use IFNγ ELIspot to identify novel immunogenic ASFV proteins and then incorporate these into viral vectors for immunization and challenge experiments in pigs.

## Results

### Screening Peptides Pools Against ASFV Immune Lymphocytes by IFNγ ELIspot

**Figure 1:**
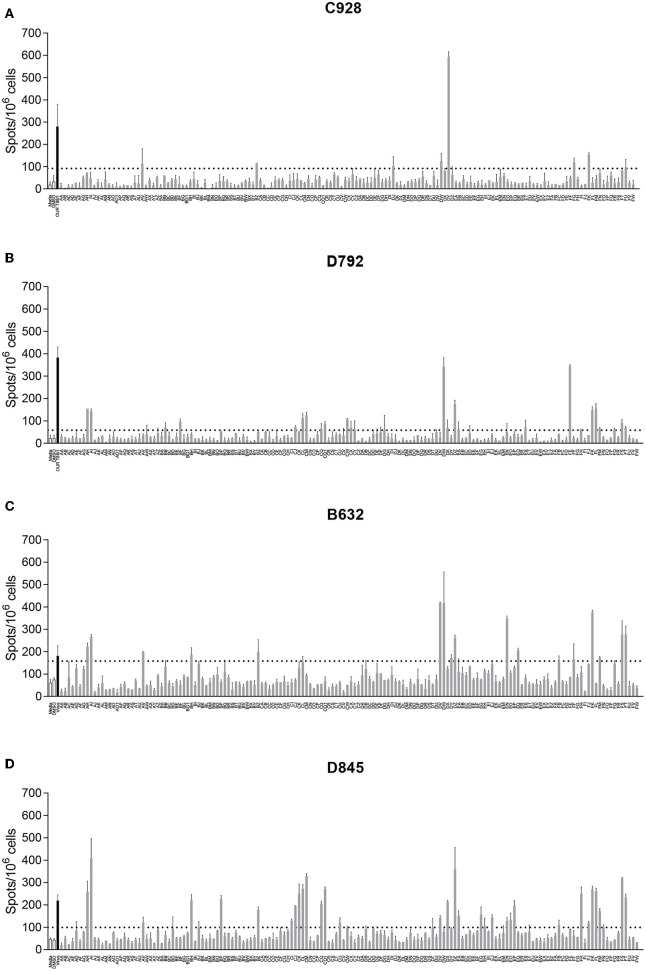
Interferon gamma (IFNγ) response to pools of peptides corresponding to ASFV open reading frames. Splenocytes from ASFV-immune pigs C931 **(A)**, D792 **(B)**, B631 **(C)**, and D847 **(D)** from Experiments 1 **(A,B)** and 2 **(C,D)** were stimulated overnight and the IFNγ response determined by ELIspot. The first two bars, outlined in black are the negative controls (media alone and DMSO), the black bar is the response to virus and responses to peptide pools AA through FW are shown in gray. The y axis shows numbers of spot forming cells detected per 10^6^ cells and x-axis indicates the stimuli. Dotted lines show two times the background plus one standard deviation.

ASFV antigens recognized by lymphocytes from ASFV-immune pigs were identified by screening pools of approximately twenty-four 20-mer peptides against splenocytes by IFNγ ELIspot. Splenocytes were derived from animals from Experiments 1 and 2 and in total five NIH *dd* minipigs, three NIH cc minipigs, and two Babraham large white pigs were screened. Splenocytes from pigs C926, C928, C931, and D792 were screened against a total of 3,647 peptides in 156 pools corresponding to 129 open reading frames (ORFs; Table S1). The remaining pigs (B631, B632, D845, D846, D847, and D848) were also screened against an additional 161 peptides in 9 pools corresponding to another 4 ORFs (Table S2). The response to a peptide pool was considered positive if there was at least double the number of IFNγ secreting cells than seen after stimulation with DMSO or media, and that the value was statistically significant (one-way ANOVA, Dunnett's multiple comparison test, *p* ≤ 0.05). An example of the IFNγ ELISpot response to different pools of peptides using cells from 4 different pigs is shown in Figure 1. Full details of the IFNγ responses to peptide pools and statistical analysis from all animals are shown in Table S3.

**Figure 2:**
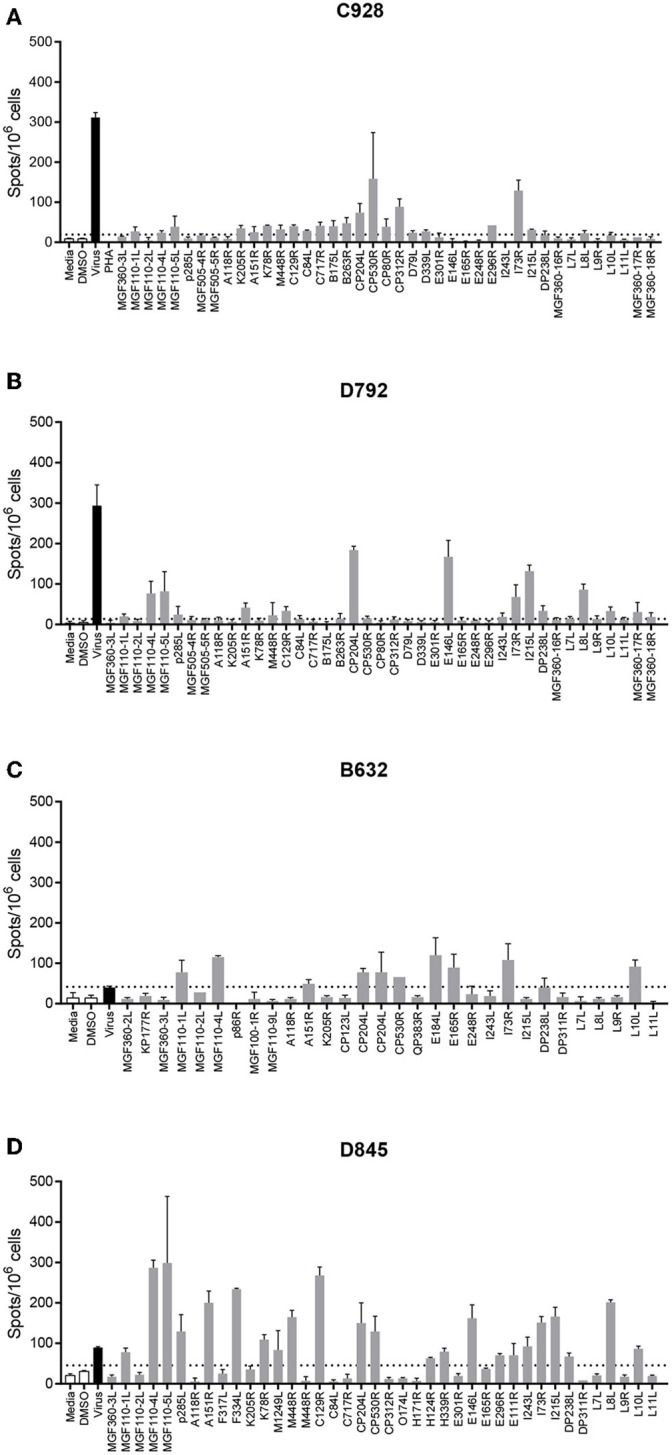
Interferon gamma (IFNγ) response to pools of peptides corresponding to individual ASFV open reading frames. Splenocytes from ASFV-immune pigs C931 **(A)**, D792 **(B)**, B631 **(C)**, and D847 **(D)** from Experiments 1 **(A,B)** and 2 **(C,D)** were stimulated overnight and the IFNγ response determined by ELIspot. The first two bars, outlined in black are the negative controls (media alone and DMSO), the black bar is the response to virus and responses to peptide pools are shown in gray. The cross-hatched bar in panel C (Pool CP204L-1), produced too many spots to count accurately. The y axis shows numbers of spot forming cells detected per 10^6^ cells and x-axis indicates the stimuli. Dotted lines show two times the background plus one standard deviation.

Most peptide pools that induced a >2-fold response above background, were composed of peptides derived from more than one ORF. Therefore, such peptide pools were broken down into subpools in which each subpool contained peptides from only one ORF. These subpools were then screened by IFNγ ELISpot using cells from the same pigs as in the previous assay (Figure 1) to determine the individual ORFs that induced secretion of IFNγ. An example of the IFNγ ELISpot responses to different subpools of peptides using cells from 4 different pigs is shown in Figure 2. Details of the IFNγ responses to peptide subpools from all animals are shown in Table S4. A range of the pools and subpools, recognized by lymphocytes from ASFV-immune pigs were also tested using PBMC purified from 7 naïve animals and no significant responses (*p* > 0.05, one-way ANOVA, Dunnett's multiple comparison test) above that seen for media or DMSO alone were observed (Supplemental Figure S1). Analysis of the data from both the pool and subpool screen revealed that peptides corresponding to 38 different proteins induced a statistically significant IFNγ response in lymphocytes from at least one pig (one-way ANOVA, Dunnett's multiple comparison test, *p* ≤ 0.05) (Table 1). Many individual peptide pools and subpools were recognized by several animals from the same inbred line, although there was variation in the magnitude of the response to a given pool or subpool between pigs. The pattern of response to the peptide library in animal D792 was similar to that of D845, D846, D847, and D848 showing consistency in the results between the two immunization and challenge experiments. Both virus and pools of peptides stimulated IFNγ secretion from CD4+CD8α+ T-cells which represent activated and effector memory T-helper cells (Supplemental Figure S2) in outbred ASF immune-pigs (9) and these double-positive T-lymphocytes are linked to protection in the OUR T88/3 model (25). IFNγ+ CD4-CD8α+ cells were also detected after incubation with virus, most of which were CD8α high and therefore likely represent cytotoxic T-cells. IFNγ-secreting cells were not detected after simulation of lymphocytes from a naïve pig with either whole virus or peptide pools. Antigen selection focused on those recognized by the *dd* minipigs as these possess a SLA haplotype that is common in the outbred population and were supplemented with antigen that induced strong IFNγ responses in ASFV-immune pigs from the other two lines. A total of 18 ORFs were selected to be taken forward for immunization and challenge experiments and are highlighted in Table 1.

**Table 1:** Pools of peptides that induced a significant IFNγ response in lymphocytes from at least one ASFV-immune pig.

	**DD Minipig^a^**		**CC Minipig^b^**		**Babraham^c^**		**Total**	
**Gene Name**	**# pigs**	**%**	**# pigs**	**%**	**# pigs**	**%**	**# pigs**	**%**
KP177R	0	0	0	0	1	50	1	10
MGF110-1L	0	0	0	0	2	100	2	20
MGF110-4L	5	100	0	0	2	100	7	70
MGF110-5L	4	80	0	0	0	0	4	40
MGF300-1L	1	20	0	0	0	0	1	10
285L	1	20	0	0	0	0	1	10
A151R	5	100	0	0	1	50	6	60
F334L	3	60	0	0	0	0	3	30
K78R	0	0	1	33.3	2	100	3	30
K205R	0	0	2	66.7	0	0	2	20
M1249L	4	80	0	0	0	0	4	40
M448R	5	100	0	0	0	0	5	50
C257L	2	40	0	0	0	0	2	20
C475L	1	20	0	0	2	100	3	30
C129R	4	80	0	0	0	0	4	40
C962R	1	20	0	0	0	0	1	10
B646L^*^	4	100	NT	NT	0	0	4	66.7
CP204L	5	100	1	33.3	2	100	8	80
CP530R	4	80	3	100	2	100	9	90
CP312R	4	80	2	66.7	2	100	8	80
O174L	1	20	0	0	0	0	1	10
NP419L	1	20	0	0	0	0	1	10
NP868R	0	0	1	33.3	0	0	1	10
H359L	0	0	0	0	2	100	2	20
H339R	1	20	0	0	2	100	3	30
E146L	5	100	0	0	0	0	5	50
E184L	0	0	0	0	2	100	2	20
E165R	0	0	0	0	2	100	2	20
E296R	2	40	0	0	0	0	2	20
E248R	0	0	0	0	1	50	1	10
I243L	1	20	0	0	0	0	1	10
I73R	5	100	3	100	2	100	10	100
I215L	5	100	0	0	0	0	5	50
DP238L	1	20	0	0	1	50	2	20
MGF360-16R	1	20	0	0	0	0	1	10
MGF505-11L	0	0	0	0	2	100	2	20
L8L	5	100	0	0	0	0	5	50
L10L	1	20	0	0	2	100	3	30

*Shaded ASFV ORFs are those selected for expression in recombinant adenovirus or MVA vectors*.

a *5 NIH dd pigs that had been infected with ASFV OUR T88/3 followed by OUR T88/1 (n = 4) or ASFV OUR T88/3 followed by OUR T88/1, and then Georgia 2007/1 (n = 1)*.

b *3 NIH cc pigs that had been infected with ASFV OUR T88/3 followed by OUR T88/1, and then Georgia 2007*.

c *2 Babraham pigs that had been infected with ASFV OUR T88/3 followed by OUR T88/1*.

* *Peptide pools corresponding to B646L were screened using cells from four NIH dd minipigs and two Babrahams that had been infected with OUR T88/3 followed by OUR T88/1. All other pools were screen against cells from all 10 pigs*.

Recombinant replication deficient adenoviruses (rAd) and modified vaccinia Ankara (MVA) expressing codon-optimized ASFV genes expressing each of the 18 selected ORFs were generated. Most of the selected genes were uncharacterized and specific antibodies were not available therefore, with the exception of CP204L, MGF110-4L, and MGF110-5L, the genes were tagged with the HA non-amer epitope to confirm gene expression by immunoblotting or immunofluorescence (Supplemental Figures S3, S4).

### Immune Responses Induced in *inbred* Pigs Immunized With a Pool of 12 rAds Expressing Individual ASFV Proteins

The protective efficacy of a pool of rAd expressing individual ASFV proteins was tested using NIH *dd* minipigs, as described in Materials and Methods, animal experiment 3. Four animals were vaccinated with 12 rAd (Antigen Pool A) divided into two pools of six different rAd. Those expressing B646L, MGF110-5L, CP204L, CP530R, I73R, and I215L were in one pool and A151R, C129R, E146L, L8L, M448R, and MGF110-4L in the other. Each of these pools were inoculated at different sites and two control animals were inoculated with an equivalent total dose of rAd expressing GFP, again in two sites. All animals were boosted 5 weeks later with the same pools of rAd at the same sites, and were challenged 5 weeks later with the virulent OUR T88/1 isolate of ASFV by the intramuscular route at a different site. No adverse reactions were observed after either the prime or boost with the pools of rAd.

**Figure 3:**
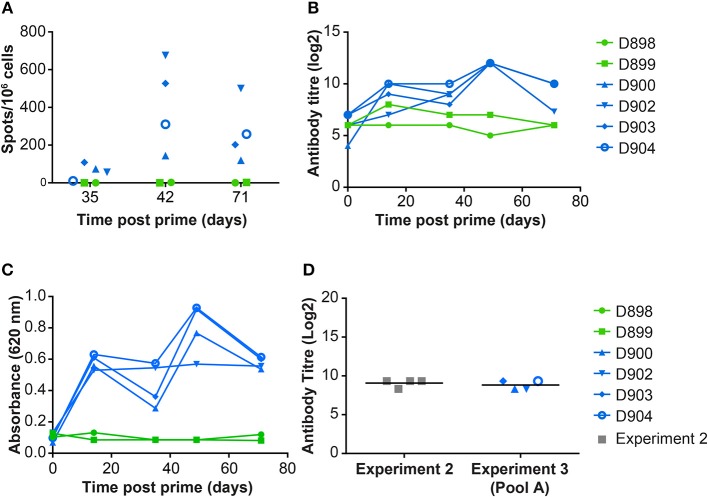
Immune responses in *dd* minipigs from Experiment 3 primed and boosted 35 days later with a pool of rAd expressing 12 individual ASFV genes. NIH *dd* mimipgs D900, D902, D903, and D904 were inoculated intramuscularly (IM) at 2 sites with 3 × 10^10^ IU of 2 pools of six rAd with each individual rAd expressing a different ASFV gene. As controls, NIH *dd* mimipgs D898 and D899 were inoculated IM at 2 sites with 3 × 10^10^ IU of rAd-GFP at each site. **(A)** Number of ASFV specific IFNγ producing mononuclear cells in the blood were enumerated by ELIspot on the indicated days post prime. Data points indicate the number of spots per million cells induced by whole virus after background subtraction of whichever was higher of the media alone or mock inoculum. **(B)** The titer of anti-ASFV antibodies in the serum of the indicated animals was determined by immunoperoxidase assay. **(C)** Antibodies to ASFV protein p30 (CP204L) were detected in serum collected from animals at the indicated times post prime by ELISA. **(D)** Antibodies to ASFV protein pp62 (CP530R) were determined by ELISA using sera from D900, D902, D903, D904 before challenge (Day 71 post prime) and, for comparison, from NIH *dd* minipigs D845, D846, D847, and D848 at termination of experiment 2 (21 days post challenge with OUR T88/1, 42 days post immunization with OUR T88/3).

Immunization with the pool of 12 rAd induced both a cellular (Figure 3A) and humoral response (Figure 3B) to whole virus. ASFV-specific IFNγ producing cells were detected in vaccinated but not control pigs, at 5, 7 and 10 weeks after vaccination. Non-specific staining of Vero cells was detected in pre-immune sera from all of the pigs. ASFV-specific serum antibody titers above background could be detected 2 weeks post prime and these peaked at a mean titer of log_2_ 12, 7 weeks post prime (Figure 3B).

**Figure 4:**
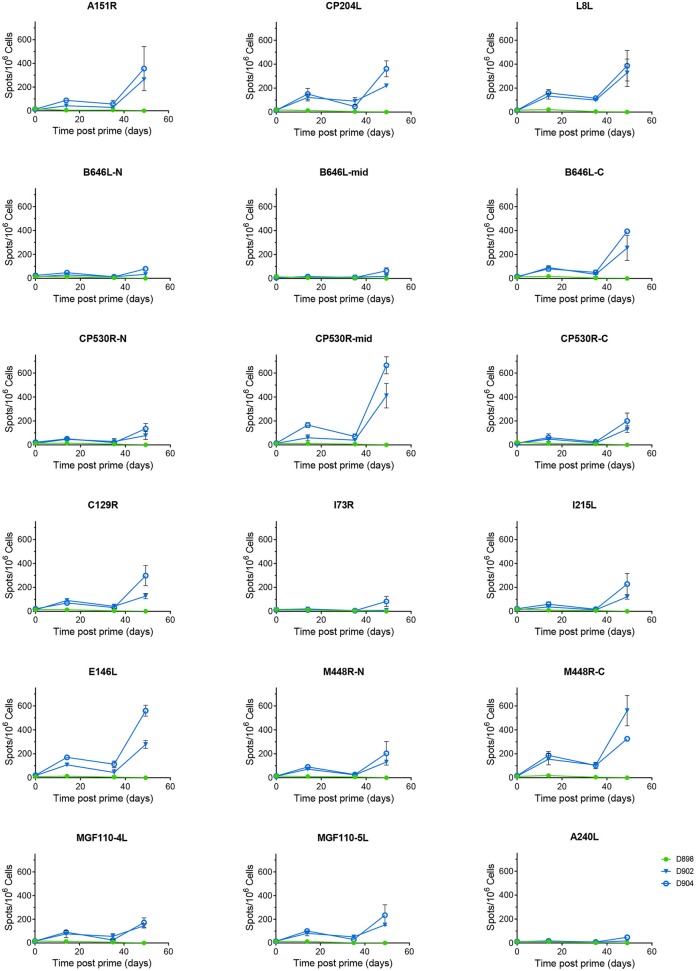
Antigen specific IFNγ responses to Antigen Pool A in NIH inbred *dd* minipigs. Animals were primed and boosted 35 days later with a pool of 12 rAd each expressing an individual ASFV gene. The number of IFNγ producing cells in the blood stimulated by media alone, 0.5% DMSO, or pools of peptides were enumerated by ELIspot on the indicated days post prime. N, mid and C indicate pools of peptides that correspond to the N-terminus, mid-section or C-terminus of the proteins encoded by B646L, CP530R, and M448R. Data points indicate the number of spots per million cells induced by the pool of peptides, error bars indicate standard deviation from the mean.

Antigen specific IFNγ secreting cells were analyzed by culturing PBMCs from animals D898, D902, and D904 with peptides corresponding to the individual ORFs expressed by the rAd and measuring numbers of IFNγ producing cells by ELISpot (Figure 4). The antibody response to these proteins was measured by ELISA (Figures 3C,D) or indirect immunostaining (Table 2). Neither an antigen-specific IFNγ response nor antibody response were detected in the control pigs vaccinated with rAd-GFP. In contract, an IFNγ response above the level induced by the control peptide pool corresponding to pA240L and carrier (DMSO) was induced to all of the proteins except for I73R in pigs immunized with rAd expressing ASFV antigens (2-way ANOVA, Dunnett's multiple comparison test). Homologous prime and boost increased the number of antigen specific IFNγ secreting cells to all of the antigens (Figure 4).

**Table 2:** Pre-challenge antibody response to individual antigens in Group A from experiment 3.

**Antigen**	**Animal number**			
	**D900**	**D902**	**D903**	**D904**
A151R	No	No	No	No
B646L	Yes^*^	Yes^*^	Yes^*^	Yes^*^
C129R	Yes	Yes	Yes	Yes
CP204L	Yes^†^	Yes^†^	Yes^†^	Yes^†^
CP530R	Yes^†^	Yes^†^	Yes^†^	Yes^†^
E146L	No	No	No	No
I73R	No	No	No	No
I215L	Yes	Yes	Yes	Yes
L8L	No	No	No	No
M448R	Yes	Yes	Yes	Yes
MGF110-4L	Yes	Yes	Yes	Yes
MGF110-5L	Yes	Yes	Yes	Yes

NIH inbred pigs were immunized and boosted with a pool of twelve rAd each expressing the indicated antigens and serum collected from the individual pigs before immunization or before challenge. Vero cells transfected with the individual antigens were stained with serum diluted 1:100, and reaction with serum antibodies was detected by indirect immunofluorescence using Alexa 488 conjugated antibodies against pig IgG. A pig was considered positive for a given antigen if the pre-immunization sera was negative and the pre-challenge sera was positive. Antigens detected by commercial competitive ELISA

* or indirect ELISA with recombinant protein

† *are shown for comparison, the latter data is shown in detail in Figure 3*.

Homologous prime and boost increased the antibody responses to CP204L, which encodes P30 (Figure 3C). Levels of anti-CP530R (pp62) antibody in the serum of pigs D900, D902, D903, and D904 at the time of ASFV challenge were similar to those seen after ASFV challenge in pigs that had been previously infected with OUR T88/3 and which had recovered from OUR T88/1 challenge (Figure 3D). Antibody responses to A151R, E146L, I73R, or L8L were not detected in any of the rAd-vaccinated pigs (Table 2). However, all of the pigs had antibodies that recognized B646L, C129R, I215L, M448R, MGF110-4L, and MGF110-5L. Therefore, with the exception of I73R, all of the antigens induced either a cellular or humoral immune response that was detectable in the blood of the animals after homologous rAd prime/boost. Antigen-specific antibodies were not detected in pre-immune sera, or in pre-challenge sera from the two control pigs, D898 and D899.

### Reduced Viral Replication and Clinical Signs in Some Pigs Immunized and Boosted With a Pool of 12 rAd Expressing Individual ASFV Genes

**Figure 5:**
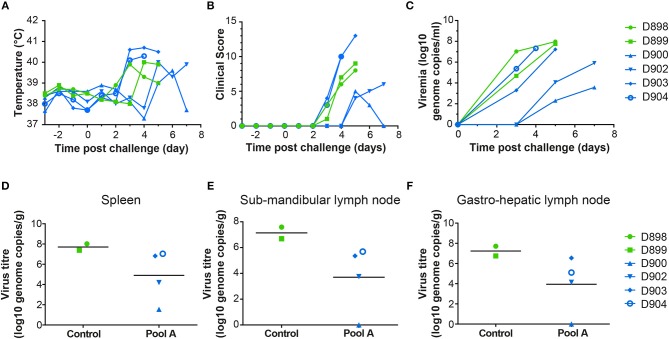
Clinical and virological parameters of NIH *dd* minipigs from Experiment 3 after ASFV challenge. Body temperature **(A)** and clinical score **(B)** of animals primed and boosted with Ad-GFP (D898 and D899) or Ad-ASFV (D900, D902, D903, and D904) are shown on the indicated days post challenge with the virulent OUR T88/1 strain of ASFV. The viral load in the blood over time **(C)** and in spleen **(D)**, sub-mandibular lymph node **(E)** and gastro-hepatic lymph node **(F)** post mortem determined by qPCR. Data points are the mean of the duplicate extractions measured in duplicate and bars indicate the mean of each group **(D–F)**.

Following ASFV challenge, the control animals (D898, D899) displayed clinical signs typical of acute ASF and were euthanized 5 days post-challenge (Figures 5A,B). Post mortem examination revealed typical lesions consistent with ASF. Levels of viremia (Figure 5C) and virus titers in tissues (Figures 5D–F) were similar to those seen in previous experiments (33, 34). There were two different responses in the animals immunized with Antigen Pool A, the two males (D903, D904) showed enhanced clinical signs compared to the controls, one animal was euthanized 4 days post-challenge (dpc) and the other 5 dpc. However, internal lesions, viremia, and viral load in tissues were slightly reduced in these pigs compared to the controls (Figure 5 and Supplemental Figure S3A). The two female pigs (D900, D902) were partially protected against ASFV, with delayed and reduced clinical signs compared to the controls. However, one of the two pigs reached its humane endpoint 7 dpc and so both animals were euthanized at this time. Although viremia and viral load in the tissues were reduced compared to the controls (Figures 5C–F), viremia increased between 5 and 7 dpc, therefore it is unclear whether viral replication was simply delayed in these animals or whether they would have recovered from infection.

At necropsy, pigs vaccinated with rAd-GFP (D898 and D899) showed macroscopic lesions characteristic of acute ASF (Supplemental Figure S3A). Moderate erythema and cyanosis was observed on skin of the ears, tail, nose and chest as well as on perianal areas where small hemorrhages were also observed. Control pigs also displayed mild hydropericardium with reddish fluid, ascites, mild hepatic congestion, petechial hemorrhages in renal cortex and moderate hyperemic splenomegaly. A generalized lymphadenitis with petechial hemorrhages was also observed in most of the lymph nodes examined. Haemorrhagic lymphadenitis was especially severe in gastrohepatic and renal lymph nodes which looked like blood clots. Pigs D904 and D903 which were euthanized 4 and 5 dpc, respectively, showing characteristic lesions of acute ASF similar to those described in non-vaccinated control pigs (hydropericardium, ascites, hepatic congestion, hyperemic splenomegaly, and haemorrhagic lymphadenitis). Both pigs displayed more severe vascular changes on the skin than described in control pigs. A generalized redness of the skin, that affected 30–40% of total skin surface, was observed along with cyanotic areas and petechial hemorrhages. Such vascular changes were observed on huge areas of skin of the ears, face, nose, neck, dorsal area of thorax, lumbar area, tail, perianal and scrotal areas, chest, abdomen, inguinal area, and limbs. On the other hand, the pigs euthanized 7 dpc (D900 and D902) showed only mild macroscopic lesions that affected mainly the spleen (hyperemic splenomegaly) and liver (congestion).

There was no correlation between the differences in viral replication and any of the measured immune responses, either to whole virus or the individual antigens. All animals had comparable serum antibody titers or numbers of ASFV antigen-specific IFNγ secreting cells in the blood (Figures 3A,B). Similarly, there was no difference in the antigens recognized by the sera from the two partially protected animals (D900 and D902) and the two animals that were not protected (D903 and D904). The antigen-specific IFNγ responses (Figure 4) detected in D902 and D904 were also very similar. The only difference between the immunized animals that had reduced viremia and those that did not was their sex, male animals showed enhanced clinical signs whereas those observed in female animals were reduced compared to the controls. Sex plays an important role in outcomes after challenge of both naïve and vaccinated hosts (35, 36), but has not been thought to play a role in disease progression in pigs infected with ASFV.

### Immune Responses Induced in Out-Bred Pigs Immunized by Heterologous rAd Prime/MVA Boosting With Pools of Vaccine Vectors Expressing Individual ASFV Genes

The results in *dd* pigs showed that the 12 selected antigens when delivered by homologous prime/boost could induce an immune response which resulted in delayed disease and reduced virus titers in some animals. Results from other animal species suggest that heterologous prime/boost induces a more robust immune response than homologous prime/boost and therefore in the next experiment we utilized a rAd prime and MVA boost with the same 12 antigens. In this experiment, vectors encoding influenza NP were used a control. In addition to repeating the pool of 12 antigens evaluated in experiment 3 (Antigen Pool A), another group of pigs were immunized with Antigen Pool B consisting of six additional antigens (CP312R, E165R, E184L, K78R, L10L, MGF110-1L) supplemented with three antigens selected at random from the original pool of 12 (A151R, E146L, MGF110-4L). Animals were immunized intramuscularly at one site, boosted 4 weeks later at the same site and then challenged 4 weeks after that with OUR T88/1 at a different intramuscular site.

**Figure 6:**
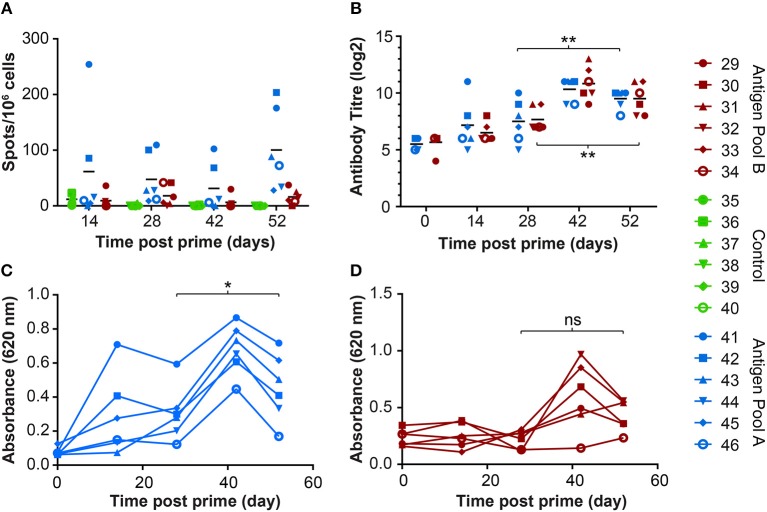
Immune responses in outbred pigs from Experiment 4 primed with the indicated pools of rAd and boosted 28 days later with MVA. **(A)** ASFV-specific IFNγ producing cells in the blood were enumerated by ELIspot on the indicated days post prime. Data points indicate the number of spots per million cells induced by whole virus after background subtraction of whichever was higher of the media alone or mock inoculum. **(B)** The titer of anti-ASFV antibodies in the serum of the indicated animals was determined by immunoperoxidase assay. Asterisks indicated significant differences (2-way ANOVA) between antibody titer pre-boost and pre-challenge for both groups of pigs immunized with Antigen Pool A (*p* = 0.0028) or Antigen Pool B (*p* = 0.0072). Antibodies to ASFV protein CP204L **(C)** or CP312R **(D)** were detected in serum collected from the indicated animals at the indicated times post prime by ELISA. Differences between O.D pre-boost and pre-challenge were analyzed using repeated measures one-way ANOVA, *p = 0.0256, ns *p* = 0.0608.

Immunization with the pools of rAd and MVAs induced both a cellular (Figure 6A) and humoral response (Figure 6B) to whole virus. There was no significant difference in the antibody response to whole virus between the two groups. However, immunization with Antigen Pool A primed ASFV-specific IFNγ-producing lymphocytes, whereas the response in pigs given Antigen Pool B was relatively poor, with the exception of animal 29. There was no significant difference between the number of ASFV-specific IFNγ-secreting cells between day 28 and day 52 in either of the two groups (2-way ANOVA), showing that the MVA boost did not increase the number of circulating virus-specific cells as has been seen in other species (37, 38). However, the MVA boost did induce a significant increase in the antibody response to whole virus (Figure 6B), as well as to CP204L in pigs given Antigen Pool A (Figure 6C).

**Figure 7:**
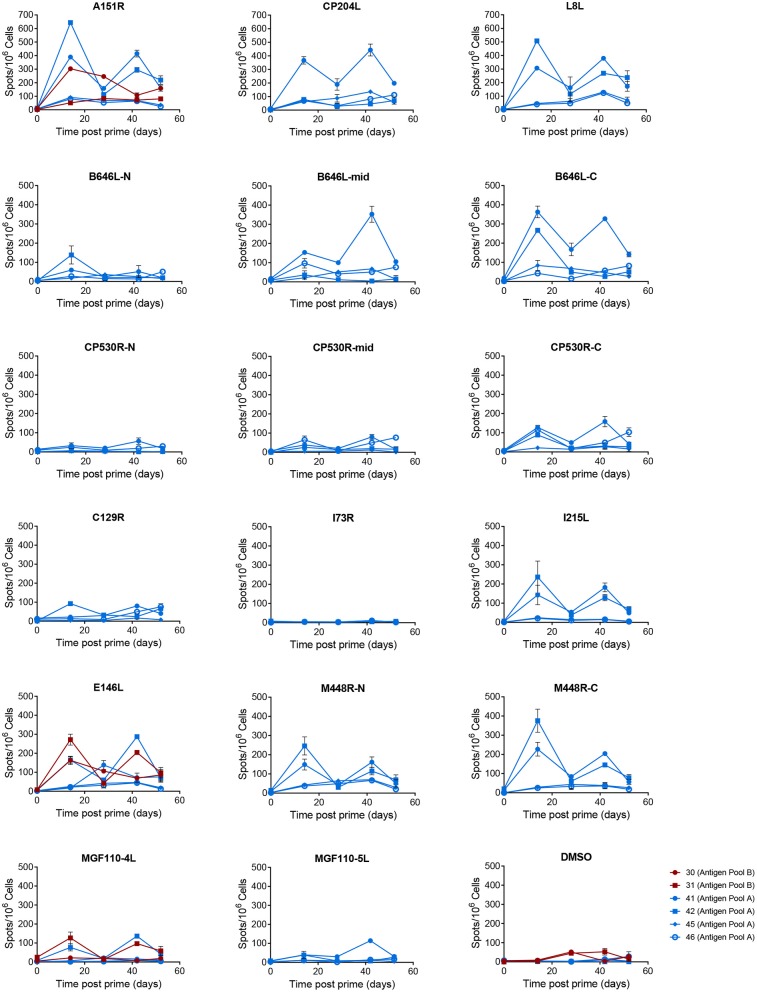
Antigen specific IFNγ responses to proteins in Antigen Pool A in outbred pigs. Animals were primed with a pool of 12 rAd (blue) or 9 rAd (red) each expressing an individual ASFV gene and then boosted 28 days later with a pool of MVA expressing the same genes. The number of IFNγ producing cells in PBMCs stimulated with media alone, 0.5% DMSO, or pools of peptides were enumerated by ELIspot on the indicated days post prime. N, mid and C indicate pools of peptides that correspond to the N-terminus, mid-section or C-terminus of the proteins encoded by B646L, CP530R, and M448R. Data points indicate the number of spots per million cells induced by the pool of peptides, error bars indicate standard deviation from the mean. Note that to aid clarity the scales for A151R, CP204L, and L8L have been set to a different range to the other pools.

**Figure 8:**
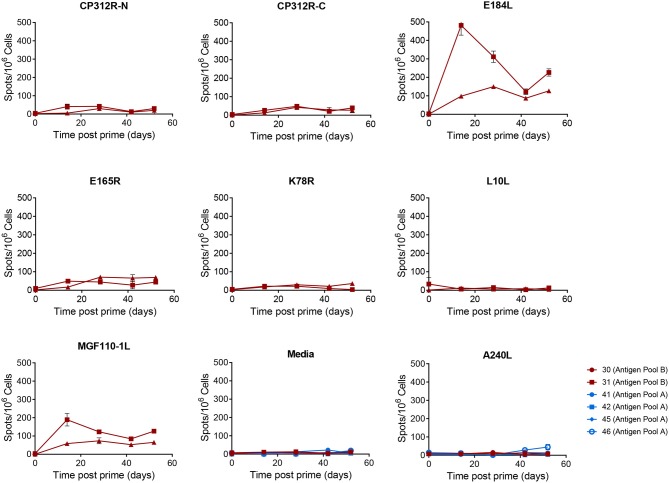
Antigen specific IFNγ responses to proteins in Antigen Pool B in outbred pigs. Animals were primed with a pool of 9 rAd each expressing an individual ASFV gene and then boosted 28 days later with a pool of MVA expressing the same genes. The number of IFNγ producing cells in the blood stimulated by media alone, 0.5% DMSO, or pools of peptides were enumerated by ELIspot on the indicated days post prime. N, mid and C indicate pools of peptides that correspond to the N-terminus, mid-section or C-terminus of the protein encoded by CP312R. Data points indicate the number of spots per million cells induced by the pool of peptides after background subtraction, error bars indicate standard deviation from the mean.

Significant numbers of IFNγ-secreting cells were detected in immunized pigs following stimulation of PBMCs with pools of peptides corresponding to most of the 18 different antigens over and above that seen in response to an irrelevant protein (pA240L) or DMSO (Figures 7, 8). No significant IFNγ response was seen after recall with peptide pools corresponding to I73R, K78R, or L10L (2-Way ANOVA, Dunnett's multiple comparison test) and a significant response after simulation with peptides from CP312R was detected only on day 42 in pig 31. Antigen-specific IFNγ responses followed similar trends to the ASFV-specific IFNγ response). Animals 41 and 42 had higher levels of antigen-specific IFNγ secreting cells than 45 and 46, with the exception of the response following stimulation with peptides from ORF CP530R where there was a comparable response in pig 46 to that seen in the other two animals. The MVA boost did not lead to higher numbers of antigen-specific IFNγ cells than those seen after the rAd prime.

Less uniformity was seen in the antigen-specific antibody responses in the outbred pigs compared to those seen in the inbred pigs from the previous experiment (Table 3). In addition to CP204L, only CP530R induced a detectable antibody response in all 6 pigs given Antigen Pool A. Antibodies recognizing C129R were identified in all of the pigs expect for animals 45 and 46. Antibodies recognizing A151R, E146L, I73R, or L8L were not detected in any of the pigs. Two pigs had antibodies that recognized I215L and M448R and a response to MGF110-4L and MGF110-5L were detected in three animals. MGF110-4L and MGF110-5L are 91.1% similar, therefore cross-reactive antibodies are quite likely. Only two pigs (pigs 41 and 42) developed antibodies that recognized protein I215L. A151R-, E146L-, K78R-, and MGF110-1L-specific antibodies were not detected in any of the animals given Antigen Pool B (Table 3). Antibodies against CP312R (Figure 6D) and E184L were detected in the serum of most of the animals, but the responses to E165R, MGF 110-4L and L10L were more variable.

**Table 3:** Pre-challenge antibody response to individual antigens in experiment 4.

**Antigen**	**Animal number**											
	**41**	**42**	**43**	**44**	**45**	**46**	**29**	**30**	**31**	**32**	**33**	**34**
A151R	No	No	No	No	No	No	No	No	No	No	No	No
B646L	No^*^	No^*^	No^*^	No^*^	No^*^	No^*^	–	–	–	–	–	–
C129R	Yes	Yes	Yes	Yes	No	No	–	–	–	–	–	–
CP204L	Yes^†^	Yes^†^	Yes^†^	Yes^†^	Yes^†^	Yes^†^	–	–	–	–	–	–
CP530R	Yes	Yes	Yes	Yes	Yes	Yes	–	–	–	–	–	–
E146L	No	No	No	No	No	No	No	No	No	No	No	No
I73R	No	No	No	No	No	No	–	–	–	–	–	–
I215L	Yes	Yes	No	No	No	No	–	–	–	–	–	–
L8L	No	No	No	No	No	No	–	–	–	–	–	–
M448R	No	Yes	No	Yes	No	No	–	–	–	–	–	–
MGF110-4L	Yes	Yes	Yes	No	No	No	Yes	Yes	Yes	Yes	Yes	No
MGF110-5L	Yes	Yes	Yes	No	No	No	–	–	–	–	–	–
CP312R	–	–	–	–	–	–	Yes^†^	Yes^†^	Yes^†^	Yes^†^	Yes^†^	No^†^
E165R	–	–	–	–	–	–	No	No	Yes	Yes	Yes	Yes
E184L	–	–	–	–	–	–	Yes	Yes	Yes	Yes	Yes	Yes
K78R	–	–	–	–	–	–	No	No	No	No	No	No
L10L	–	–	–	–	–	–	No	No	Yes	Yes	Yes	No
MGF110-1L	–	–	–	–	–	–	No	No	No	No	No	No

Outbred pigs were immunized with pools of rAd each expressing the indicated antigens and then boosted with MVA. Animals 41 through 46 were primed and boosted with vectors expressing Antigen Pool A and animals 29 through 34 with Antigen Pool B. Serum was taken from the individual pigs before immunization or before challenge. Vero cells transfected with the individual antigens were stained with serum diluted 1:100, and reaction with serum antibodies was detected by indirect immunofluorescence using Alexa 488 conjugated antibodies against pig IgG. A pig was considered positive (indicated by Yes) for a given antigen if the pre-immunization sera was negative and the pre-challenge sera was positive. Antigens detected by commercial competitive ELISA

* or indirect ELISA with recombinant protein

† *are shown for comparison, the latter data is shown in detail in Figures 6C,D*.

### Differing Outcomes After Challenge of Out-Bred Pigs Immunized by Heterologous rAd Prime/MVA Boosting With Pools of Vaccine Vectors Expressing Individual ASFV Proteins

**Figure 9:**
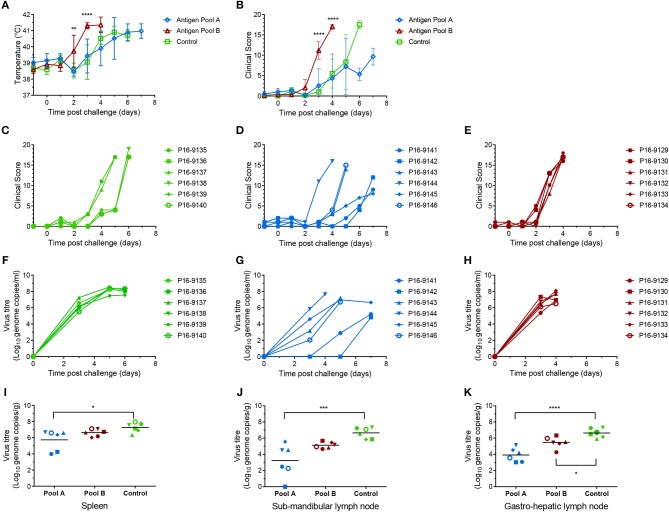
Clinical and virological parameters of immunized outbred pigs after ASFV challenge. Mean temperatures **(A)** and clinical scores **(B)** of groups of six pigs primed and boosted with Antigen Pool A (blue), Antigen Pool B (red) or a control antigen (green). Asterisks on panels A and B show significant differences between the mean temperatures or clinical scores, respectively, of the group of pigs immunized with influenza NP and Antigen Pool B (2-way ANOVA on data from day−1 to day 4). Clinical scores for each individual pig immunized with influenza NP **(C)**, Antigen Pool A **(D)** or Antigen Pool B **(E)**. Blood was taken from animals immunized with Influenza NP **(F)**, Antigen Pool A **(G)**, Antigen Pool B **(H)** on the indicated days. Spleen **(I)**, sub-mandibular lymph node **(J)** and gastro-hepatic lymph node **(K)** taken post mortem were extracted in duplicate and the viral titer determined by qPCR, with each extraction tested in duplicate. Data points are the mean of the duplicate extractions. Bars on panels I to K indicate the mean of each group and asterisks indicate significant differences (ordinary one-way ANOVA) between Control and either Antigen Pool A or Pool B. Errors bars on panels A and B indicate standard deviation from the mean, *p ≤ 0.05, **p ≤ 0.01, ***p ≤ 0.001 or ****p ≤ 0.0001.

Animals immunized with rAd and MVA expressing influenza NP developed clinical signs typical of the early stages of ASF after challenge (Figures 9A–C) and were euthanized 5 and 6 dpc. Animals immunized with Antigen Pool B showed early clinical signs (Figures 9B,E), including temperatures of 41.8 and 41.5°C in animals 29 and 30, 2 days post-challenge (Figure 9A). All animals in this group showed recumbancy and inappetence 3 dpc and were euthanized 4 dpc (Figures 9B,E). Clinical signs and temperatures in the pigs immunized with Antigen Pool B were significantly higher than the controls 3 and 4 days post challenge (*p* < 0.0001, two-way ANOVA with Tukey's multiple comparison test) suggesting immunization resulted in enhanced diseased after challenge. Pigs immunized with Antigen Pool A exhibited a varied response after challenge with ASF (Figure 9D). Pig 44 showed enhanced clinical signs similar to those seen for the pigs given Antigen Pool B and was euthanized 4 dpc, two others were euthanized 5 dpc, having a similar clinical picture to the controls. However, three of the pigs presented reduced clinical signs up until 7 dpc when they reached the humane endpoint of the experiment. Pigs immunized with Antigen Pool B had viral titers in the blood that were similar to those seen in the controls (compare Figures 9F,H). Viral load in the tissues were slightly reduced compared to the controls (Figures 9I–K), however the pigs given Antigen Pool B were euthanized 4 dpc whereas the controls reached their humane end-points on 5 and 6 dpc. With the exception of animal 44, the pigs immunized with Antigen Pool A had lower levels of viraemia 3 dpc compared to the controls (Figure 9G). Pigs 41 and 42 had much lower levels of viraemia than the other animals 3 dpc, and virus titers were approximately 100 to 1,000-fold less than the controls at termination. Interestingly pig 45 had similar levels of viremia to pigs 43 and 46 at 5 dpc, but did not exhibit clinical signs typical of ASF. At termination of the experiment, there were also significant differences in viral loads in the tissues between animals immunized with Antigen Pool A and the control animals (Figures 9I–K). As seen with Experiment 3, rAd prime and MVA boost with a pool of vectors expressing twelve antigens induced a delay in clinical signs and reduced viral replication in some animals. Viremia increased between 5 and 7 dpc in animals 41 and 42 given Antigen Pool A, therefore it is unclear whether vaccination simply delayed the onset of ASF or whether there was reduced replication that the animals could have recovered from.

Macroscopic evaluations revealed the presence of macroscopic lesions characteristic of acute forms of ASF (39, 40). All vaccinated pigs given Antigen Pool B were euthanized 4 dpc, showing the same characteristic macroscopic lesions present in control pigs euthanized between 5 and 6 dpc (Supplemental Figure S3). Most of the pigs included in these groups displayed skin erythema and cyanosis on the skin of the ears (12/12), mild hydropericardium (11/12), mild ascites (12/12), moderate to severe hyperemic splenomegaly (9/12) as well as haemorrhagic lymphadenitis (11/12) that affected mainly gastrohepatic (7/12) and renal lymph nodes (11/12). Some pigs also showed petechial hemorrhages in kidney (3/12). Some lesions observed in pigs in the control group, but not in pigs immunized with Antigen Pool B, were subcutaneous hemorrhages (1/6), mild hepatic congestion (2/6) and mild pulmonary interstitial oedema (1/6).

Pigs immunized with Antigen Pool A were euthanized between 4 and 7 dpc. In those pigs euthanized at 4 and 5 dpc, macroscopic lesions were similar to those described in most of pigs given Antigen Pool B and the controls (mild hydropericardium, and ascites, mild to severe hyperemic splenomegaly and haemorrhagic lymphadenitis). On the other hand, together with the typical ASF lesions, the three pigs euthanized at 7 dpc (pigs 41, 42, and 45) also showed lesions associated to more intense vascular changes, such as mild to moderate pulmonary interstitial oedema (3/3), moderate pulmonary congestion (1/3) and moderate hepatic congestion (2/3), likely as a consequence of a longer clinical course.

In contrast to findings from Experiment 3 there were clear correlations between the induced immune responses and the clinical signs and viremia after challenge. Pigs 41 and 42 which were immunized with Antigen Pool A had the highest number of ASFV-specific IFNγ secreting cells before challenge suggesting a relationship between lower levels of viral replication and the magnitude of the cellular response induced by this pool of antigens. Pigs 41 and 42 were also the only two pigs to develop antibodies to I125L. All of the animals immunized with Antigen Pool B, which induced a humoral response, but a weak cellular response to whole virus, developed enhanced disease compared to the control group. Pigs 44 and 45 that were immunized with Antigen Pool A had ASFV-specific antibody and cellular responses comparable to those immunized with Antigen Pool B, however pig 44 was the only animal in this group that developed enhanced disease and pig 45 showed clinical signs similar to pig 41 and 42 that had reduced viremia. Therefore, the disease enhancement seen after challenge of pigs immunized with Antigen Pool B is likely a consequence of the specific antigens in the pool rather than an ASFV-specific antibody response *per se*.

### Relationship Between Experimental Outcomes and SLA Genotypes

NIH *dd* pigs are homozygous for SLA class I haplotype Lr-4.0 and class II haplotype Lr-0.4 (referred to as Lr-4.4), NIH *cc* pigs are Lr-3.3 and Babraham pigs are Lr-55.6 (41). Immunogenicity of the antigens in outbred pigs could be dependent on them possessing the SLA alleles from the inbred animals that the antigens were originally selected from. Likewise, the differences between the cellular immune response and protective efficacy induced by Antigen Pool A could be linked to the SLA haplotype. Protection against virulent ASFV after immunization with the low virulent strain OUR T88/3 has also been linked to SLA haplotype (3). Twelve different SLA class I haplotypes and 11 different SLA class II haplotypes were identified in the outbred pigs used in Experiment 4 (Table S5). Of these, class I haplotype Lr-4.0 was the most common and was detected in six animals, followed by Lr-24.0 which was detected in four animals. The most common class II haplotype was Lr-0.23 which was detected in six animals and Lr-0.14 and Lr-0.19a each in three. There was little similarity in the SLA alleles between the animals that had reduced clinical signs and viraemia (pigs 41 and 42), and those encoded by the NIH *dd* inbred pigs from which Antigen Pool A were primarily derived. Other than the SLA-3^*^04:XX allele encoded by pig 41 and the DQA^*^02:XX allele encoded by pig 42, none of the other SLA alleles were shared by the *dd* inbred pigs. Furthermore, animals 44 and 46 were not protected and both of these animals possessed the same class I haplotype Lr-4.0 as the NIH *dd* pigs, with pig 46 being SLA class I identical (homozygous) to *dd*, while pig 44 also shared the same class II alleles of DRB^*^02:XX and DQA^*^02:XX as the *dd*. Taken together, this indicates that the reduced viraemia in the two outbred pigs given Antigen Pool A was not dependent on the presence of SLA-I or SLA-II haplotypes encoded by the NIH *dd* minipigs. Interestingly pigs 41 and 42 shared SLA-1^*^08:XX, SLA-2^*^05:XX and DQA^*^01:XX alleles, however only SLA-1^*^08:XX was common to the two animals with reduced viremia and absent from the remaining outbred animals immunized with Antigen Pool A. MGF110-1L, E165R, E184L, and L10L that were included in Antigen Pool B were selected based on their immunogenicity in the Babraham line of pigs bearing the haplotype Lr-55.6. Cellular responses to E184L and MGF110-1L were detected in both pigs 30 and 31 and the only shared allele between these animals and the Babraham line was DQA^*^01:XX. Taken together the data suggested antigen-specific cellular immune responses in outbred pigs were not dependent on those pigs possessing the same SLA genotypes as the inbred pigs used to initially identify the antigens.

## Discussion

Peptide library screening of splenocytes from ASFV immune pigs from three different strains of inbred pigs significantly increases our knowledge of the breadth of the immune response to ASFV infection. Previous studies have shown that p72 (29) and p30 (28) are recognized by cytotoxic lymphocytes from ASFV-immune pigs. We have shown that peptides from these proteins are also recognized by IFNγ secreting cells by lymphocytes from ASFV-immune pigs, and have identified a further 36 ORFs from which pools of peptides stimulate IFNγ-producing cells primed by infection with the OUR T88/3 strain of the virus. Two peptides corresponding to the 25 aa cytotoxic T-cell epitope previously identified in the major capsid protein p72 (29) did not induce detectable secretion of IFNγ (pool BG1), although incubation of splenocytes derived from animals from Experiment 2 with peptides corresponding to the C-terminal third of p72 (pool GB) induced high numbers of IFNγ secreting cells in *dd* animals. Peptide pools induced IFNγ secretion from the same CD4+CD8α+ T-cell subset that has been linked to protection in pigs immunized with OUR T88/3. Whether these peptide pools contain CD4 or CD8 epitopes remains to be determined, however they are unlikely to recognized by γδ cells as these are predominately CD4- (42). All of the genes we identified have been previously shown to be expressed in cultured cells infected with OUR T88/3 stain of ASFV (43) or in pigs infected with the Georgia 2007/1 strain of ASFV (44). We excluded 28 predicted ORFs from our screen which were predominately late proteins, as experiments with vaccinia virus suggest that antigens recognized by CD8^+^ T cells are predominately encoded by early genes (45, 46). As seen with vaccinia virus, our results show that a diverse set of ASFV proteins can induce secretion of IFNγ from lymphocytes in pigs that have recovered from ASF, but not from lymphocytes of naïve pigs (45–50). It is likely that there are additional antigens within the 28 proteins omitted from the screen, however by analogy with what has been described for vaccinia virus these may be CD4 antigens (49) and DNA vaccination that induced CD4 responses to ASFV proteins were not protective (51). Another important caveat is the lack of reliable functional genomic data for ASFV; a previous study suggested that sequences outside of the predicted ORFs can induce proliferation of lymphocytes from ASFV-immune animals (31) and many of the ASFV proteins represented by the peptide library are based on predictions rather than experimental data.

Eighteen different viral proteins were incorporated into viral vectors and used to immunize pigs which were subsequently challenged with virulent ASFV. Although there was evidence of partial protection against ASFV in a proportion of immunized pigs, they all developed both a cellular and humoral immune response to whole virus. Numbers of ASFV specific-IFNγ secreting cells induced by immunization with the viral vectors in some of the pigs were comparable to those seen in other studies using live attenuated viruses (4, 5, 52). Although the number of ASFV specific IFNγ secreting cells correlated with reduced viraemia in outbred pigs immunized with the pool of 12 antigens in experiment 4, they did not correlate with reduced viremia in the NIH inbred minipigs in Experiment 3. This discrepancy is consistent with other studies suggesting that the number of ASFV specific-IFNγ secreting cells in the blood is not a reliable correlate of protection (5–7, 27). Detailed analysis of the lymphocyte subsets that secrete IFNγ and other cytokines, mediate ASFV-specific cytotoxicity, or those which proliferate in response to virus may help to determine if aspects of the cellular immune response correlate with the reduced viraemia seen in these experiments. Importantly we also show that antigens identified in an inbred pig with a defined SLA haplotype can induce an immune response in outbred pigs expressing a diverse selection of haplotypes. There was no commonality between the SLA haplotypes of the inbred pig lines used to identify the antigens and those outbred animals that either had a cellular immune response to the antigens or that were partially protected after challenge.

### Immunization With Viral Vectors Induce ASFV and Antigen Specific Antibody Responses

Although the approach here was to generate a cellular immune response to ASFV, viral vectors are effective inducers of antigen-specific antibody responses (37, 38, 53), and therefore we analyzed both virus and antigen-specific antibody responses in the immunized pigs. Anti-ASFV antibody titers determined by fixed cell ELISA were similar to those seen in pigs by immunization with rationally attenuated viruses lacking the B119L (9GL) (27, 52) or B119L and DP96R (UK) genes (7). However, the animals immunized with these attenuated viruses were protected from challenge with related virulent ASFV, whereas the pigs immunized with viral vectors in the experiments described here were not. Determining the importance of the antibody response in protection is complicated by the lack of a functional assay with which to dissect it, as we were unable to detect infection enhancing or neutralizing activity in the serum of any of the animals. Future experiments could test for antibody-dependent cellular cytotoxicity as has been described in serum from pigs recovered from ASFV (22). It is unlikely that the serum from the animals contain antibodies capable of inhibiting haemadsorption as our pools of antigens did not include the CD2v (EP402R) or EP153R proteins that are involved in mediating this phenomenon (54, 55).

Other studies have shown that A151R, in a cocktail of six different vectors encoding seven different antigens (one of the antigens was a fusion) (16), has induced antibody titers as high as 1:51200 by indirect ELISA against recombinant protein, whereas we were unable to detect an antibody response to this protein by fixed cell-ELISA in any of the 16 vaccinated pigs. This discrepancy could be due to the different experimental approach used to detect A151R-specific antibodies. Immunization of four NIH *dd* minipigs with rAd expressing CP530R induced similar antibody titers to those seen in animals protected from OUR T88/1 challenge by immunization with the OUR T88/3 strain, but were several orders of magnitude lower than those reported by others in pigs immunized with a cocktail of virus-vectored ASFV genes (15). However, the latter study used a 10- to 20-times greater dose of each rAd with an adjuvant by the latter. Antibody responses to K78R, E184L, and CP312R have been identified in domestic swine that have recovered from ASFV infection (18, 19) and we were able to detect antigen specific responses to viral vectored E184L and CP312R, but not K78R. L10L encodes for a homolog of the highly antigenic p22 protein that is immunogenic in swine when delivered as a recombinant protein (10), however only 50% of the pigs immunized with viral vectored L10L developed a detectable antibody response to the protein. We were also unable to detect an immune response to the I73R protein despite peptides from this protein inducing secretion of IFNγ from all three inbred pig lines. As immunogenicity *in vivo* did not appear to correlate with expression levels *in vitro*, it is likely that the vaccine platform may need tailoring to the individual protein in certain cases.

### Dissecting the Viral Vectored Immune Response to ASFV

Similar to previous experiments (15) pools of rAd induced robust immune responses in swine to many of the antigens in the viral vector pools, particularly within Antigen Pool A. The MVA boost significantly increased the antibody response, but an increase in the cellular immune response above that seen after priming was not detected. Experiments with macaques (37) and humans (38) have shown >2-fold increases in the cellular immune response using rAd-prime/MVA-boost regimes. The MVA dose used here was proportionally similar to that used in humans (7.5 × 10^7^ pfu in ~40 kg pigs, 2 × 10^8^ pfu per 75 kg human in the UK), but less than that used in macaques (1 × 10^8^ pfu per 7.5 kg animal). Therefore it is possible that increasing the dose of MVA may increase the efficacy of the boost with respect to the cellular immune response, although inoculation with significantly less MVA (1 × 10^7^ TCID_50_ per pig) has induced antigen specific immune responses in pigs (17). No adverse post-immunization reactions were observed in any of the animals suggesting that doses up to 6 × 10^10^ IU rAd and 8.75 × 10^8^ pfu MVA were well tolerated in pigs.

Secretion of IFNγ by lymphocytes from pigs immunized with Antigen Pool B in response to whole virus was poor compared to the response to a number of the individual antigens within the pool. Peptides corresponding to A151R, CP312R, E146L, E184L, and MGF110-1L all induced >100 spots per million cells in pig 30 (Figure 8) on day 14 whereas whole virus induced 33 spots. Antigen Pool A also included A151R and E146L and immunization of pigs D902, D903 in Experiment 3 and 41 and 42 in Experiment 4 with this pool induced ASFV-specific responses as well as responses to both A151R and E146L. Taken together this suggests that the immune response induced by viral vectored expression of A151R and E146L did not to contribute to the observed secretion of IFNγ to ASFV.

Previous experiments have shown that DNA vaccination with a plasmid encoding the CP204L and E183L genes can induce enhanced virus replication which was linked to antibody dependent enhancement *in vitro* (51). Antigen Pool A induced enhanced disease, but not enhanced virus replication in two male NIH *dd* inbred minipigs, whereas Antigen Pool B induced clear enhanced disease in all six female outbred pigs. There is no common antigen shared between the two experiments presented here and previous work. CP204L was present in Antigen Pool A and the previous study (51) whereas A151R, E146L, and MGF110-4L are shared between Antigen Pool A and Antigen Pool B. Therefore, it is likely that the immune response to at least two different ASFV proteins are capable of disease enhancement. L10L is a homolog of the internal envelope protein p22 and CP312R, E146L, and E184L have recently been identified as components of the ASFV virion (56). Due to the absence of a detectable cellular response in the animals immunized with Antigen Pool B it is likely that enhanced disease is due to the induced antibody response and it is tempting to speculate that these novel virion proteins may be involved. The difference in outcome after challenge between the male and female NIH *dd* inbred pigs was striking. Sex is a determinant in protection against HSV-1 and HSV-2 after vaccination in humans (57) and in protection against heterologous strains of influenza virus in mice (58). Passive transfer of antibodies from female mice can protect males against homologous influenza drift variants, but not vice versa and is dependent on TLR7 (59). No differences were seen in the antibody titers to the antigens that were tested between male and female NIH *dd* minipigs immunized with the viral vectors, however not all antigens were tested therefore we cannot conclude that the antibody response was not responsible for the observed differences between the sexes. African swine fever disease enhancement is not dependent on sex *per se* as all of the animals immunized with Antigen Pool B were female.

In conclusion, we have identified more than thirty ASFV proteins that are recognized by lymphocytes from ASF immune pigs; significantly increasing our knowledge of the determinants of the cellular immune response to ASFV. Vaccination of pigs with a subset of these proteins vectored by rAd and MVA induced an ASFV-specific immune response, which reduced viremia in a proportion of inbred and outbred pigs. Strengthening this immune response, possibly through the use of adjuvants, additional proteins not screened in these experiments, different combinations of the proteins identified here, or in other studies (12, 14) could lead to protection from disease and a route to an effective ASF subunit vaccine.

## Materials and Methods

### Antibodies

Monoclonal antibody C18 against p30 (CP204L) and rabbit anti-XP124L (MGF110-4L) have been described previously (60). Rat anti-HA monoclonal antibody 3F10 was purchased from Roche, mouse anti-V5 monoclonal antibody SV5-PK1 was from BioRad, and mouse anti-porcine IFNγ monoclonal antibodies P2F6 and P2C11 were from Thermo-Fisher Scientific.

### Viruses and Cells

Tissue cultured adapted Ba71v, low virulent OUR T88/3, virulent OUR T88/1 and virulent Georgia 2007/1 ASFV strains have been described previously (33, 61, 62). OUR T88/3, OUR T88/1 and Georgia 2007/1 viruses were grown and titrated on bone marrow cells prepared from the femurs of 4 to 6 week old Large White outbred pigs, Ba71v was grown on Vero cells. Bone marrow cells were cultured for 3 days in EBSS (Sigma) supplemented with 4 mM HEPES, 10% heat-inactivated porcine serum (BioSera) and 100 IU/ml penicillin and 100 μg/ml streptomycin in plastic multi-well plates or culture flasks prior to infection. Vero cells were maintained in DMEM-HEPES supplemented with 10% heat-inactivated fetal calf serum and 100 IU/ml penicillin and 100 μg/ml streptomycin. Mock inoculum was prepared from uninfected cell cultures. Virus titers were determined by end point dilution using the Spearman-Karber method as the amount of virus causing haemadsorption in 50% of infected cultures (HAD) or as the amount of virus causing 50% of cells to stain positive for ASFV early protein p30 by immunofluorescence (infectious units (IU). PBMC and splenocytes were cultured in RMPI, GlutaMAX, HEPES supplemented with 10% fetal calf serum, 1 mM sodium pyruvate, 50 μM 2-mercaptoethanol, 100 IU/ml penicillin and 100 μg/ml streptomycin (RPMI/10).

### Recombinant Vectors

ASFV open reading frames (ORFs) were codon-optimized for expression in *Sus scrofa*, synthesized and cloned into pcDNA3.1zeo(+) (Thermo Fisher). All of the ORFs except for CP204L, I73R, MGF110-4L and MGF110-5L were synthesized with an HA tag at the 3′-end. I73R was synthesized with an HA tag at the 5′-end and CP204L and MGF110-4L and−5L were not tagged. ASFV ORFs were then sub-cloned into transfer plasmids for making recombinant replication deficient human adenovirus 5 (rAd) and recombinant modified vaccinia Ankara (MVA) using standard techniques. Purified viral vectors were generated by the Jenner Institute Viral Vector Core Facility (Oxford) (63, 64). The MVA transfer plasmids, and hence the final viral vectors, also contained GFP under the control of a viral promoter to enable rapid plaque purification of positive clones. Vectors expressing GFP and influenza NP have been described previously (65).

A plasmid containing the V5 epitope sequence was created by ligating overlapping oligonucleotides into restriction endonuclease digested pcDNA3.1zeo(+). Codon-optimized ASFV genes were then amplified by PCR and sub-cloned in frame with the V5 epitope to create C-terminally tagged expression plasmids and confirmed by sequencing.

### Swine Leucocyte Antigen (SLA) Genotyping

Genotyping of three SLA class I (*SLA-1, SLA-2, SLA-3*) and three SLA class II (*DRB1, DQB1, DQA*) genes was performed using low-resolution PCR-based assays with sequence-specific typing primers as previously described (66, 67). Modifications were made to the typing primer panels to broaden the allele coverage with the increasing number of SLA alleles (CS Ho, unpublished data). SLA haplotypes were deduced based on the comparison with published haplotypes (66, 68–70) and unpublished haplotypes identified in various commercial and experimental pig populations (CS Ho, unpublished data).

### Animal Experiments and Ethics Statement

All of the animal experiments were carried out under the Home Office Animals (Scientific Procedures) Act (1986) (ASPA). Four animal experiments were approved by the Animal Welfare and Ethical Review Board (AWERB) of The Pirbright Institute. The immunization steps of Experiment 4 were carried out at APHA Weybridge and were approved by the Animal and Plant Health Agency AWERB. The animals were housed in accordance with the Code of Practice for the Housing and Care of Animals Bred, Supplied or Used for Scientific Purposes, and bedding and species specific enrichment were provided throughout the study to ensure high standards of welfare. Through careful monitoring, pigs that reached the scientific or humane endpoints of the studies were euthanized by an overdose of anesthetic. All procedures were conducted by Personal License holders who were trained and competent and under the auspices of Project Licenses.

An overview of these experiments is presented in Table 4. SLA-defined NIH *cc* and *dd* minipigs (71) and inbred Large white Babraham pigs (41, 72) were obtained from herds kept at The Pirbright Institute, Compton. Female Landrace × Large white pigs were obtained from a high health farm in the UK. Scoring of clinical signs and macroscopic lesions assessed at post-mortem were as described (4, 39).

**Table 4:** Overview of animals experiments showing immunogens used for prime, boost, challenge, and the genetic background of the pigs.

**Experiment**	**1**	**2**	**3**	**4**
Pig breeds	*cc* minipig *dd* minipig	Babraham *dd* minipig	*dd* minipig	Outbred
Animal Numbers	D792, C926, C928, and C931	B631, B632, D845, D846, D847, and D848	D898, D899, D900, D902, D903, and D904	29 through 46 (18 pigs)
Prime	OUR T88/3	OUR T88/3	rAd	rAd
Boost	OUR T88/1	OUR T88/1	rAd	MVA
Challenge	Georgia 2007/1	None	OUR T88/1	OUR T88/1

#### Experiment 1

One NIH *dd* inbred pig (animal number D792) and three NIH *cc* inbred pigs (C926, C928 and C931) weighing between 15 and 20 kg were inoculated intramuscularly in the rump with 10,000 IU of OUR T88/3 and challenged with the same amount of virulent ASFV, OUR T88/1, 3 weeks later. Three weeks later, the animals were challenged with 10,000 HAD of Georgia 2007/1. Inoculation of C926, C928 and D792 with OUR T88/3 as well as challenge of unvaccinated control animals with Georgia 2007/1 has been described previously (73). The animals were euthanized 3 weeks after inoculation with Georgia 2007/1.

#### Experiment 2

Two Babraham (animal numbers B631, B632) and four NIH *dd* inbred pigs (D845, D846, D847 and D848) weighing between 15 and 20 kg were inoculated via the intramuscular route with 10,000 IU of OUR T88/3. Three weeks later the animals, along with two control Babraham pigs (B635 and B636) were challenged with 10,000 HAD of OUR T88/1. Surviving animals were euthanized 3 weeks after inoculation with OUR T88/1.

#### Experiment 3

Two NIH *dd* minipigs (D898 and D899) were inoculated in each rump with 3 × 10^10^ IU of rAd-GFP. Four NIH *dd* minipigs (D900, D902, D903, and D904) were inoculated intramuscularly in either rump with 2,pools of six rAd with each individual rAd expressing a different ASFV gene. Each rAd was administered at a dose of 5 × 10^9^ IU. Pigs were between 13 and 18 weeks old. Pigs were immunized in one rump with rAds expressing B646L-HA, MGF110-5L, CP204L, CP530R-HA, HA-I73R, and I215L-HA and in the other rump with rAds expressing A151R-HA, C129R-HA, E146L-HA, L8L-HA, M448R-HA, and MGF110-4L. Five weeks later the pigs were inoculated with the same rAd in the same sites and 5 weeks after that the animals were challenged intramuscularly in the neck with 10,000 HAD OUR T88/1.

#### Experiment 4

Groups of six outbred pigs were inoculated intramuscularly in the neck with rAd expressing the influenza nucleoprotein (PR8 strain) (Control, pigs 35 to 40), a pool of twelve rAd expressing ASFV ORF (Antigen Pool A, pigs 41 to 46) or a pool of nine rAd expressing ASFV ORFs (Antigen Pool B, pigs 29 to 34). Antigen Pool A consisted of all of the antigens used in Experiment 3, i.e., B646L-HA, MGF110-5L, CP204L, CP530R-HA, HA-I73R, I215L-HA, A151R-HA, C129R-HA, E146L-HA, L8L-HA, M448R-HA and MGF110-4L. Antigen Pool B consisted of MGF110-1L-HA, K78R-HA, CP312R-HA, E165R-HA, E184L-HA, L10L-HA, A151R-HA, E146L-HA, and MGF110-4L. Each rAd was administered at a dose of 5 × 10^9^ IU, therefore animals given NP Control and Antigen Pool A were immunized with a total of 6 × 10^10^ IU rAd per pig and Antigen Pool B with a total of 4.5 × 10^10^ IU rAd. Four weeks later the pigs were inoculated in the same site with MVAs expressing the same ORFs. Each MVA expressing an ASFV gene was administered at a dose of 7.5 × 10^7^ pfu, except for L8L which was used at 5 × 10^7^ pfu. Therefore, animals given NP Control and Antigen Pool A were immunized with a total of 8.75 × 10^8^ pfu MVA and Antigen Pool B were immunized with 6.75 × 10^8^ pfu of MVA per pig. Four weeks later the animals were challenged by the intramuscular route in the rump with 10,000 HAD OUR T88/1.

### Interferon Gamma (IFNγ) ELISpot

Peripheral blood mononuclear cells (PBMC) were purified from anti-coagulant blood using histopaque gradients and splenocytes were prepared by forcing shredded tissue through muslin. Both PBMC and splenocytes were then washed extensively with PBS. The response to ASFV was analyzed using fresh cells, however the response to peptides was analyzed using cells that had been frozen, where viability was ≥90% after thawing. PVDF membrane multiwell plates (Millipore, MAIPS4510) were coated overnight at 4°C with 4 μg/ml anti-porcine IFNγ (P2F6) in 0.5 M carbonate-bicarbonate coating buffer and then washed with PBS. Cells were plated in duplicate at two different dilutions, typically 5 × 10^5^ and 2.5 × 10^5^ per well in RMPI/10. Cells were then incubated overnight in a final volume of 200 μl with media alone, 0.5% DMSO, 10^5^ HAD of OUR T88/1 or an equivalent volume of mock inoculum, or 2.5 μg/ml PHA, or peptide pools. 20 mer peptides overlapping by 10 amino acids were supplied at 1 to 3 mg scale (Mimotopes). The maximum amount of peptides in any one pool was 26 and therefore there was at most 78 μg of peptides per well, with each individual peptide being at a final concentration of 5 to 15 μg/ml. The molecular mass of the peptides varied between 1737.86 and 2758.11, therefore the final molarity of the peptides varied between 1.8 and 8.6 μM. Cells were lysed by incubating for 5 min in water and then washed with PBS. Biotinylated anti-porcine IFNγ (P2C11), followed by streptavidin conjugated to alkaline phosphatase was used to visualize spots which were then counted using an ELIspot Reader System (AID). The number of spots was converted into the number of spots per million cells and the mean of duplicate wells plotted. In experiments where the number of IFNγ secreting cells were measured over time, the response to background (the highest of media/mock/DMSO) was subtracted from the response to whole virus or peptide—this is indicated in the figure legends.

### Fixed-Cell ELISA

Anti-ASFV antibody titers were determined using an immunoperoxidase assay, by incubating 2-fold serial dilutions of sera on Ba71v infected Vero cells fixed 16 h post infection with 4% paraformaldehyde (74). Cells were permeabilized with 0.2% Triton X-100, blocked with 5% milk in PBS-0.05% Tween 20 for 1 h, then incubated with diluted sera for another hour and finally with protein A HRP conjugate. Cells were washed five times with PBS-0.05% Tween20 between each step. Positive wells were identified by AEC staining (2 mM 3-amino-9-ethylcarbazole and 0.015% H_2_O_2_ diluted in 50 mM sodium acetate buffer). All pigs showed non-specific background staining of uninfected cells after staining with day 0 sera at dilutions varying from 1:16 to 1:128.

The presence of antibodies to individual proteins was determined using indirect immunofluorescence. Sera were used at a single dilution of 1:100 and incubated with Vero cells transfected with each of the individual genes fused to a V5 tag except for MGF 110-4L and MGF 110-5L, for which a specific serum was available. Pre-immunization and pre-challenge sera were tested from pigs immunized with the pools of viral vectors expressing ASFV ORFs. Only pre-challenge sera from pigs immunized with the control antigens was analyzed for antibodies to ASFV proteins. Mouse anti-V5 tag (AbD Serotec MCA1360) diluted 1:1,000 was used as a positive control for V5 fusion gene expression. In indirect immunofluorescence, the anti-V5 antibody or a rabbit serum raised against MGF 110-4L (anti-pXP124L), were incubated simultaneously with the animal sera on transfected cells. Secondary antibodies were goat anti-mouse IgG (H+L)-AF594 (Life technologies) or donkey anti-rabbit AF594 (Life technologies) and goat anti-porcine IgG (H+L)-AF488 (Southern Biotech), all added at a dilution of 1:1,000 to the cells. The cells were then observed under the fluorescence microscope and screened for simultaneous green and red fluorescence, resulting from recognition by specific antibodies in the pig serum of the expressed viral protein and the presence of the V5 tag, respectively.

### Protein Purification

Transformed *E. coli* cells were grown overnight in selective medium. The culture was diluted 1:50 in fresh Luria–Bertani medium and grown for 2 h. Expression of recombinant proteins was induced with 1 mM IPTG for 2 h and cells were harvested by centrifugation at 7,500 rpm in a Sorvall SLA-1500 rotor for 10 min. Proteins were purified using RediPack GST Purification Modules (Amersham) according to the manufacturer's protocol.

### Indirect ELISA (p30 and CP312R)

Polysorp ELISA plates (Nunc) were coated with ASFV recombinant proteins p30 and CP312R (50 μl per well) diluted (1–10 μg/ml) in coating buffer (50 mM sodium carbonate/bicarbonate buffer, pH 9.6) and incubated overnight at 4°C. The wells were then washed three times with PBS plus 0.05 % Tween 20 and blocked with PBS plus 5 % milk (200 μl per well) at 37°C for 1 h. After blocking, plates were washed five times as above and incubated for 1 h at 37°C with pig sera diluted 1:100 in PBS plus 5 % milk (50 μl per well). The plates were again washed five times and incubated with protein A–horseradish peroxidase (Pierce) diluted 1:5,000 (100 μl per well) for 1 h at 37 °C. Finally, plates were washed again and developed with 3-dimethylaminobenzoic acid/3-methyl-2-benzothiazolinone hydrazine hydrochloride/H_2_O_2_ dissolved in 0.1 M phosphate buffer. After stopping the reaction with 3 M H_2_SO_4_ (50 μl per well), the absorbance at 620 nm was read on a Cytation3 microplate reader (Biotek).

### Indirect ELISA—CP530R (pp62)

Briefly, microtiter plates were incubated at 4°C overnight with 50 μl/well with 0.3 μg of pp62 recombinant antigens using coating buffer (0.1 M carbonate buffer, pH 9.6). The coated plates were washed with PBS-T (PBS, pH 7.5, containing 0.05% [v/v] Tween 20) and used immediately or stored at −20°C until use. The plates were subsequently blocked with PBS-TM (PBS, pH 7.5, 0.05% [v/v] Tween 20, 3% [w/v] skim milk), and duplicate samples of porcine sera were tested at a 1:20 dilution in PBS-TM by incubation for 1 h at 37°C. Positive and negative reference sera were included on each plate. HRPO-labeled protein A was added diluted 1:3,200 in PBS-TM, and the plates were incubated for 1 h at 37°C. After the plates were washed, 0.2 ml of 7,12-dimethyl-1,2-benz[a]anthracene (DMBA)−3-methyl-2-benzothiazolinone hydrazone (MBTH) substrate (Sigma) was added per well. The reaction was stopped by the addition of 50 μl of 3 N H_2_SO_4_, and the optical density at 620 nm (OD620) was measured after incubation for 10 min at room temperature.

## Author Contributions

CN, LD, and GT contributed to the conception and design of the study. CN, LCG, AR, RP, RHN, SM, LG, RN, VN, CG, C-SH, PS-C, and GT executed the study. CN wrote the first draft of the manuscript. CG, PS-C, and C-SH, wrote sections of the manuscript. All authors contributed to manuscript revision, and read and approved the submitted version.

### Conflict of Interest Statement

The authors declare that the research was conducted in the absence of any commercial or financial relationships that could be construed as a potential conflict of interest.
