# Supplementary material for: Identification and Immunogenicity of African Swine Fever Virus Antigens
Source: Front Immunol. 2019 Jun 19;10:1318. doi: 10.3389/fimmu.2019.01318 (PMC6593957; doi:10.3389/fimmu.2019.01318)
Supplement: Supplementary file 3 [file Data_Sheet_1.PDF]

Supplementary Material

Identification and immunogenicity of African swine fever virus antigens

Christopher L Netherton[1]*, Lynnette C Goatley[1], Ana Luisa Reis[1], Raquel Portugal[1], Rachel H. Nash[1], Sophie B. Morgan[1], Lynden Gault[2], Raquel Nieto[3], Veronica Norlin[2], Carmina Gallardo[3], Chak-Sum Ho[2], Pedro J. Sánchez-Cordón[1†], Geraldine Taylor[1], Linda K Dixon[1]

Supplementary Methods

Antibodies

Target	Clone/Name	Species	Dilution
HA	3F10	Rat	1:1000
ASFV P30	C18	Mouse	1:1000
ASFV XP124L	R29	Rabbit	1:1000

Flow cytometry

PBMC were defrosted, counted and 5 × 10^5 cells per well allowed to rest overnight in 200 µl RMPI, at in 96 U bottomed plates. The following morning, media was replaced and cells were incubated for 6 hours with media alone, 0.5% DMSO, 2 × 10^5 HAD of OUR T88/1 or an equivalent volume of mock inoculum, or peptide pools as described above for ELIspot. Fresh media supplemented with Golgi-Plug was then added to the cells, along with phorbol 12-myristate 13-acetate (100 ng/ml) and ionomycin (2µg/ml) to the positive controls. After a further 4 hours incubation, cells were stained for flow cytometry using Zombie NIR, anti-CD3 PE-Cy7 (BB23-8E6-8C8; 1:50), anti-CD4 PerCP-Cy5.5 (74-12-4; 1:50), and anti-CD8α FITC (MIL12; 1:100), and then fixed with Cytofix-Cytoperm. Intracellular IFNγ was detected in the presence of Cytoperm-Wash with anti-IFNγ biotin (P2C11; 1:500) followed by streptavidin BV650. Stained cells were analysed on a BD LSR Fortessa.

Protein expression analysis

Vero cells or porcine bone marrow cultures were infected with 125 IU/cell of rAd or 10 pfu/cell MVA encoding ASFV genes. Cells were fixed with 4% paraformaldehyde for 30 minutes or lysed in sample preparation buffer 20 hour post infection (hpi) with MVA and 48 hpi with rAd. Cells for immunofluorescence were permeabilised with 0.2% Triton X-100 in PBS, incubated with blocking buffer (50 mM Tris pH 7.4, 150 mM NaCl, 0.2% (w/v) gelatin, 10% (v/v) normal goat serum). Primary and secondary antibodies were also diluted in blocking buffer, cells were washed with PBS between all stages.
Protein lysates were resolved with 10% bis-tris gels, transferred to PVDF membranes, blocked with 5% milk powder and then probed overnight with primary antibody diluted in 5% BSA. Secondary antibodies were diluted in 5% milk powder, all solutions were based on TBS containing 0.2% Tween 20. Bands were detected by enhanced chemiluminescence (Pierce) with either X-ray film or with a Syngene G-box.

Supplementary Tables

Table S1: Open reading frames screened against all pigs. List of open reading frames represented in the peptide library and the number of peptides per open reading frame.

ORF	Number of Peptides	ORF	Number of Peptides	ORF	Number of Peptides
KP93L	5	K205R	20	D339L	33
MGF360-1L	36	K78R	7	D205R	20
MGF360-2L	36	K196R	19	D345L	24
KP177R	17	EP424R	42	S183L	18
L83L	8	EP152R	15	H359L	35
L60L	6	EP47L‡	4	H171R	17
MGF360-3L	40	EP364R	36	H124R	12
MGF110-1L	26	M1249L	124	H339R	33
MGF110-2L	10	M448R	44	H108R	10
MGF110-4L	12	C129R	12	H233R	23
MGF110-5L	12	C84L	8	H240R	23
285L	9	C717R	71	R298L	29
86R	8	C122R	10	Q706L	70
MGF100-1R	12	C257L	25	QP509L	50
MGF110-9L	27	C475L	47	QP383R	38
MG110-13L	15	C315R	31	E184L	18
MG110-14L	12	C147L	14	E183L	18
MGF360-4L	38	C62L	6	E301R	30
X69R	6	C962R	96	E146L	14
MGF300-1L	26	B119L	11	E165R	16
X64R	6	B169L	17	E248R	24
MGF300-2R	15	B646L†	2	E296R	29
MGF300-4L	32	B475L	47	E111R	11
MGF360-8L	31	B354L	35	E66L	6
MGF360-9L	33	B125R	12	I267L	26
MGF505-3R	10	B117L	11	I243L	24
MGF505-4R	50	B407L	41	I73R	7
MGF505-5R	49	B175L	17	I215L	21
MGF505-7R	52	B263R	26	DP238L	23
MGF505-8R	52	B66L	6	MGF360-16R	31
MGF505-9R	50	G1340L	133	MGF505-11L	54
MGF505-10R	54	G1211R	121	MGF100-2L	12
A240L	23	CP123L	12	MGF100-3L	14
A118R	11	CP204L	19	L7L*	10
A151R	15	CP530R	52	L8L*	10
MGF360-15R	27	CP80R	7	L9R*	9
A238L	23	CP312R	31	L10L	16
A859L	85	O174L	17	L11L	7
F317L	31	NP419L	41	MGF360-17R	7
F334L	33	NP868R	86	MGF360-18R	14
F778R	77	D250R	24	DP71L	7
F165R	16	D129L	12	DP96R	9
F1055L	105	D79L	7	MGF360-19R	30

*L7L, L8L and L9R have been mistakenly referred to as I7L, I8L and I9R. The original description of these genes used lowercase letter l which has been transcribed into uppercase i in

the annotation of some ASFV genomes, note that L7L and L8L were originally assigned as members of MGF100 (1).
†Two 20mers overlapping by 15 aa corresponding to the peptide identified by Leitão *et al.*, 1998 (2).
‡Minor ORF initiating at position 60004 of the OUR T88/3 genome.

Table S2: Open reading frames screened against pigs in Experiment 2. Additional open reading frames screened against splenocytes from B631, B632, D845, D846, D847 and D848 only. List of open reading frames represented in the peptide library and the number of peptides per open reading frame.

ORF	Number of Peptides
A179L	17
C44L*	4
B646L	64
E423R	43

* Minor ORF identified as a serological determinant in pigs recovered from infection with the moderately virulent Malta strain of ASFV (3).

Table S3: IFNγ ELIspot response to peptide pools corresponding to the indicated open read frames. Tables show name of peptide pool, open reading frame(s) within the pool, mean, standard deviation and number of replicates for each pool. The fold difference increase over DMSO or media alone and a p value of the difference between the individual pool and DMSO or media alone (Ordinary one-way ANOVA, Dunnett's multiple comparison test) is shown for each pool.

Table S4: IFNγ ELIspot response to peptide pools corresponding to individual open reading frames. Tables show the name of the pools in Table S3 from which the open reading frame was derived, the mean, standard deviation and number of replicates for each open reading frame. The fold difference increase over DMSO or media alone and a p value of the difference between the individual pool and DMSO or media alone (Ordinary one-way ANOVA, Dunnett's multiple comparison test) is shown for each open reading frame.

Table S5: SLA genotypes and haplotypes of pigs used in Experiments 3 and 4. Animals D900, D902, D903 and D904 from Experiment 3 were immunised with Antigen Pool A, Animals P16-9129 through P16-9134 from Experiment 4 with Antigen Pool B, and animals P16-9141 through P16-9146 from Experiment 4 with Antigen Pool A. SLA genotypes of three class I genes; SLA-1, SLA-2 and SLA-3 and three class II genes; DRB1, DQB1 and DQA were determined from each pig by PCR-SSP typing. SLA genotypes are shown as low-resolution allele groups (e.g. SLA-1*04:XX), intermediate-resolution allele strings (e.g. DRB1*04:03-04) or specific high-resolution alleles (e.g. DQB1*02:01), for example pig P16-9131 bears the SLA-1*14:01 allele and one of the SLA-1*04:XX group alleles; up to two different alleles from the SLA-3*04:XX group; and one allele from the SLA-2*04:XX group and one from the SLA-2*06:XX. The inferred class I and class II SLA haplotype is also shown for each pig, with the high-resolution haplotype data for the NIH *cc*, *dd* and Babraham (Bab) shown for reference.

**Exp. Group**	**Pig ID**	**SLA I allele specificity**			**SLA II allele specificity**			**Inferred Haplotype**		
		**SLA-1**	**SLA-3**	**SLA-2**	**DRB1**	**DQB1**	**DQA**	**Class I**	**Class II**	**Complete**
3A	D900	04:XX	04:XX	04:XX	02:XX	04:XX	02:XX	4/4	4/4	4.4/4.4
	D902	04:XX	04:XX	04:XX	02:XX	04:XX	02:XX	4/4	4/4	4.4/4.4
	D903	04:XX	04:XX	04:XX	02:XX	04:XX	02:XX	4/4	4/4	4.4/4.4
	D904	04:XX	04:XX	04:XX	02:XX	04:XX	02:XX	4/4	4/4	4.4/4.4
4A	41	08:XX,16:03	04:XX,07:XX	05:XX,06:XX	04:03-04,10:XX	06:XX,07:XX	01:XX,03:XX	7/24	19a/23	7.23/24.19a
	42	07:03,08:XX	05:XX,06:01	05:XX,10:XX	04:XX,06:XX	02:02,03:XX	01:XX,02:XX	21/26	15b/20	21.15b/26.20
	43	07:XX,09:XX,15:XX	05:XX,07:XX	05:XX,09:XX	05:XX,10:XX	06:XX,08:XX	01:XX	28/37	6/23	28.23/37.6
	44	04:XX	04:XX,05:XX	04:XX,10:XX	02:XX,10:XX	02:01,06:XX	01:XX,02:XX	4/39	2/23	4.2/39.23
	45	16:03	04:XX	06:XX	04:03-04,06:XX	07:XX	01:XX,03:XX	24/24	12a/19a	24.12a/24.19a
	46	04:XX	04:XX	04:XX	01:XX,09:XX	01:XX,08:XX	01:XX,03:XX	4/4	1/14	4.1/4.14
4B	29	12:XX,13:01,16:03	04:XX,05:XX	06:XX,10:XX	04:03-04,10:XX	06:XX,07:XX	01:XX,03:XX	24/35	19a/23	24.19a/35.23
	30	08:XX	05:XX,06:01	10:XX,12:XX	04:XX,10:XX	02:02,06:XX	01:XX,02:XX	22/39	15b/23	22.15b/39.23
	31	04:XX,14:01	04:XX	04:XX,06:XX	06:02,09:XX	07:XX,08:XX	01:XX,03:XX	4/62	12a/14	4.14/62.12a
	32	04:XX,12:XX,13:01	04:XX,05:XX	04:XX,10:XX	02:XX,04:03-04	03:02-03,04:XX	02:XX	4/35	4/13	4.4/35.13
	33	01:XX,12:XX,13:01	01:XX,05:XX	01:XX,10:XX	01:XX,04:03-04	01:XX,03:02-03	01:XX,02:XX	1/35	1/13	1.1/35.13
	34	04:XX,08:XX	04:XX,07:XX	04:XX,05:XX	09:XX,10:XX	06:XX,08:XX	01:XX,03:XX	4/7	14/23	4.14/7.23
cc	Ref	Null	03:01	03:01	03:01	03:01	01:02	3/3	3/3	3.3/3.3
dd	Ref	04:01:01	04:01	04:01	02:01:01	04:01:01	02:02:01	4a/4a	4a/4a	4a.4a/4a.4a
Bab	Ref	14:02	04:03	11:04	05:01	08:01	01:03	55/55	6/6	55.6/55.6

Supplementary Figures

Figure S1: Interferon gamma (IFNγ) response to pools of peptides corresponding to ASFV open reading frames. Splenocytes from naïve pigs CC A, CC B, D728, D731, B628, D849 or D850 were stimulated overnight with the indicated pools of peptides and the IFNγ response determined by ELIspot. The first two bars, outlined in black are the negative controls (media alone and DMSO), the black bar is the response to virus and responses to peptide pools are shown in grey. The hatched bar shows the response to PHA which in animals CC A, CC B, D728 and D731 were too numerous to count and have been set arbitrarily at 200 spots per million cells. The y axis shows numbers of spot forming cells detected per 10^6 cells and x-axis indicates the stimuli.

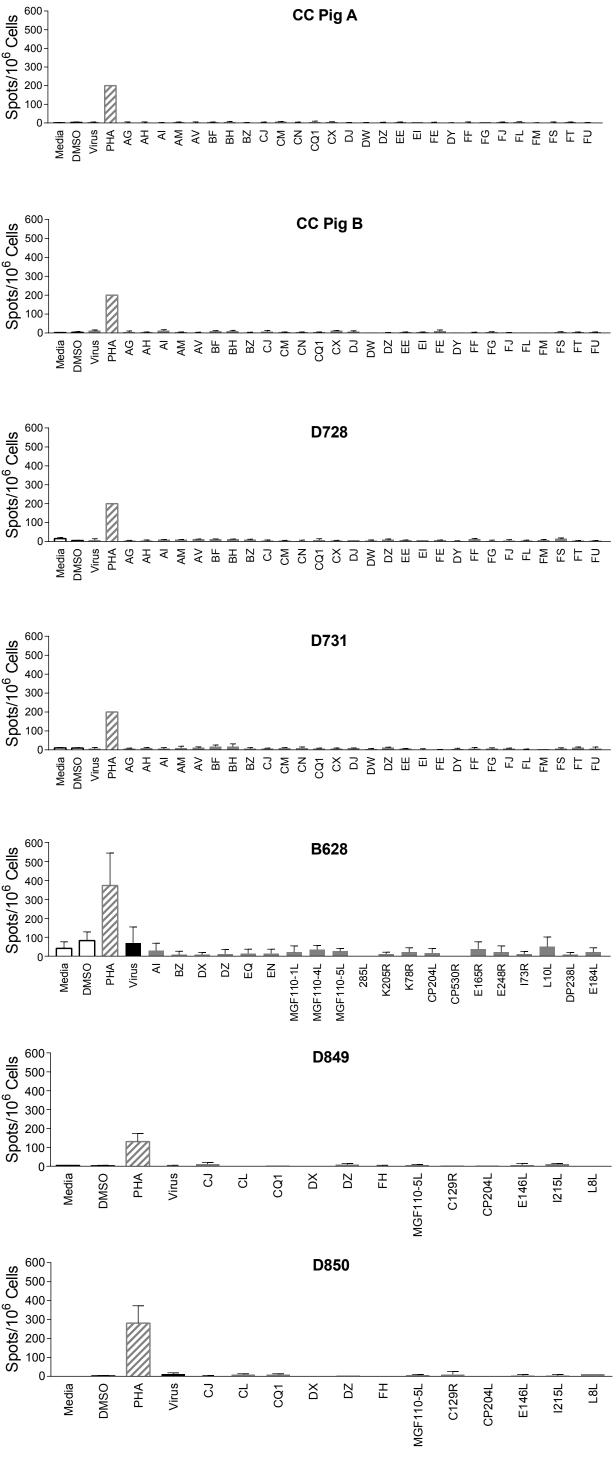
CC Pig A
Spots/10^6 Cells
600 500 400 300 200 100 0
Media DMSO Virus PHA AG AH AI AM AV BF BH BZ CJ CM CN CQ1 CX DJ DW DZ EE EI FE DY FF FG FJ FL FM FS FT FU
CC Pig B
Spots/10^6 Cells
600 500 400 300 200 100 0
Media DMSO Virus PHA AG AH AI AM AV BF BH BZ CJ CM CN CQ1 CX DJ DW DZ EE EI FE DY FF FG FJ FL FM FS FT FU
D728
Spots/10^6 Cells
600 500 400 300 200 100 0
Media DMSO Virus PHA AG AH AI AM AV BF BH BZ CJ CM CN CQ1 CX DJ DW DZ EE EI FE DY FF FG FJ FL FM FS FT FU
D731
Spots/10^6 Cells
600 500 400 300 200 100 0
Media DMSO Virus PHA AG AH AI AM AV BF BH BZ CJ CM CN CQ1 CX DJ DW DZ EE EI FE DY FF FG FJ FL FM FS FT FU
B628
Spots/10^6 Cells
600 500 400 300 200 100 0
Media DMSO PHA Virus AI BZ DX DZ EQ EN MGF110-1L MGF110-4L MGF110-5L 285L K205R K78R CP204L CP530R E165R E248R I73R L10L DP238L E184L
D849
Spots/10^6 Cells
600 500 400 300 200 100 0
Media DMSO PHA Virus CJ CL CQ1 DX DZ FH MGF110-5L C129R CP204L E146L I215L L8L
D850
Spots/10^6 Cells
600 500 400 300 200 100 0
Media DMSO PHA Virus CJ CL CQ1 DX DZ FH MGF110-5L C129R CP204L E146L I215L L8L

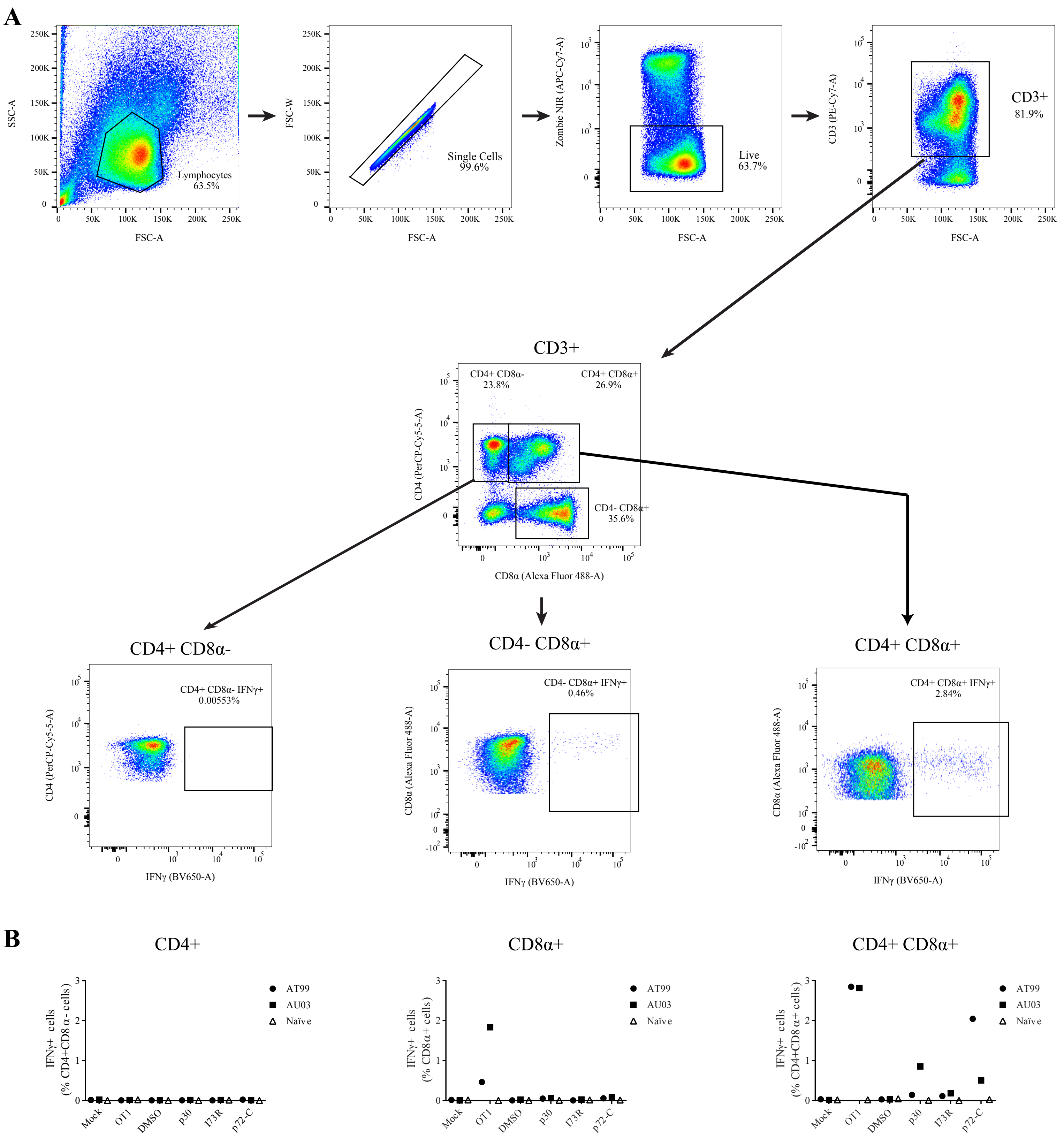

Supplemental Figure S2: Phenotype of lymphocytes secreting IFNγ. PBMC from two pigs immunised with OUR T88/3 and challenged with Benin 1997/1 (AT99 and AU03) and one naive pig were incubated with media containing OUR T88/1, a mock control, DMSO or pools of peptides corresponding to CP204L (p30), I73R or the C terminus of p72 (B646L) for six hours and then for a further four hours in the presence of brefeldin A (GolgiPlug). Cells were stained for CD3, CD4, CD8α and IFNγ and the percentage of CD3+ subsets positive for IFNγ determined by flow cytometry. **A)** Gating strategy using cells from pig AT99 stimulated with OUR T88/1 as an example. **B)** Percentage of selected populations of lymphocytes positive for IFNγ after incubation with the indicated conditions. OT1 - OUR T88/1 strain of ASFV.

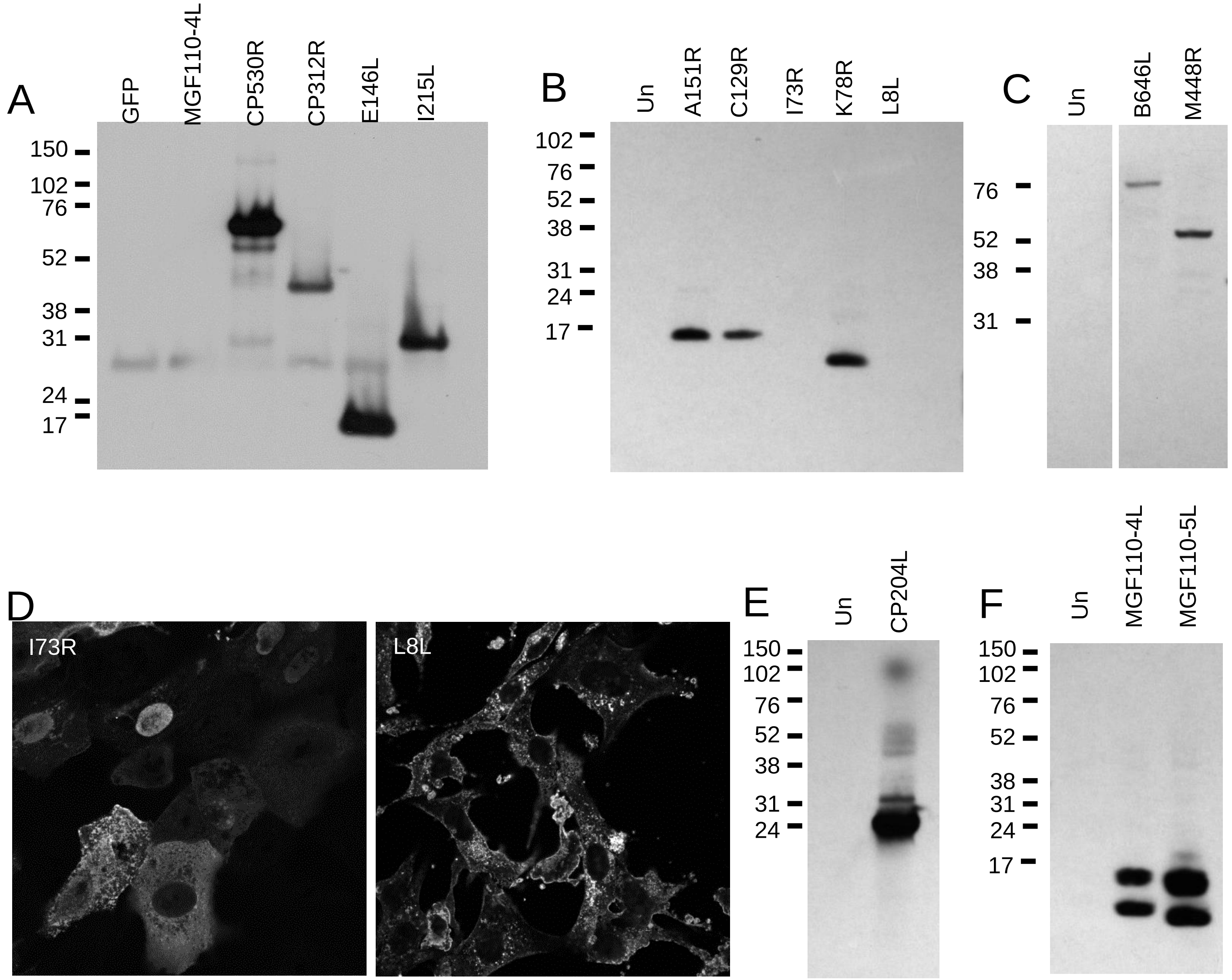

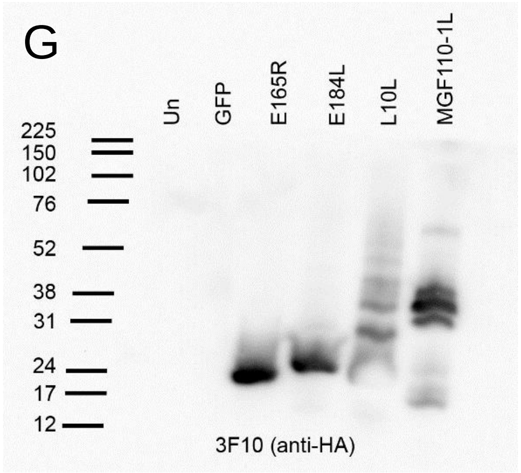

Supplemental Figure S3: Adenovirus driven expression of ASFV genes. Cells were transduced with replication deficient human adenovirus 5 expressing the indicated ASFV genes or GFP and then either lysed (Panel A-C, E-G) or fixed (D) 48 hours later. Gene expression was determined by immunoblot or indirect immunofluorescence using anti-HA clone 3F10 (A-D), anti-ASFV p30 clone C18 (E) or rabbit anti-XP124L serum R29 (F).

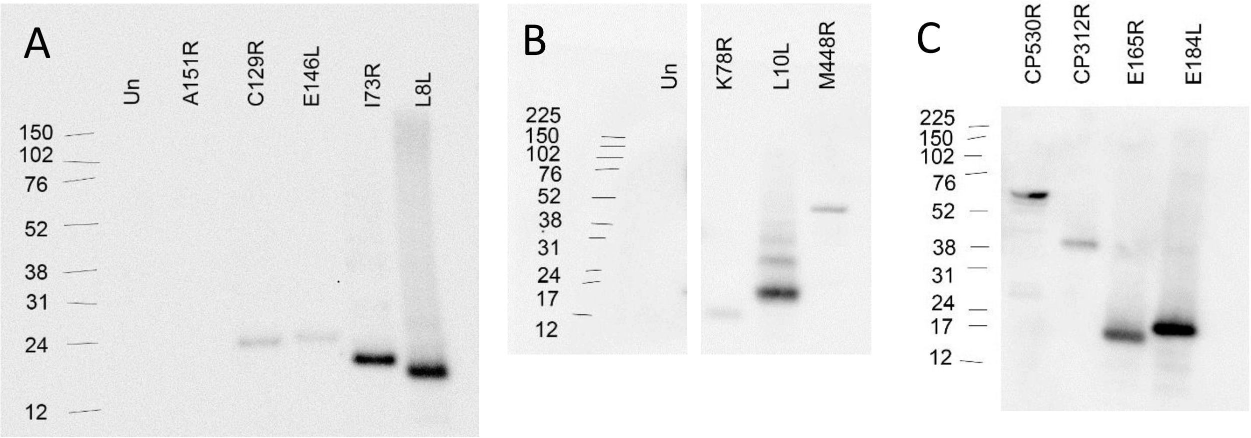

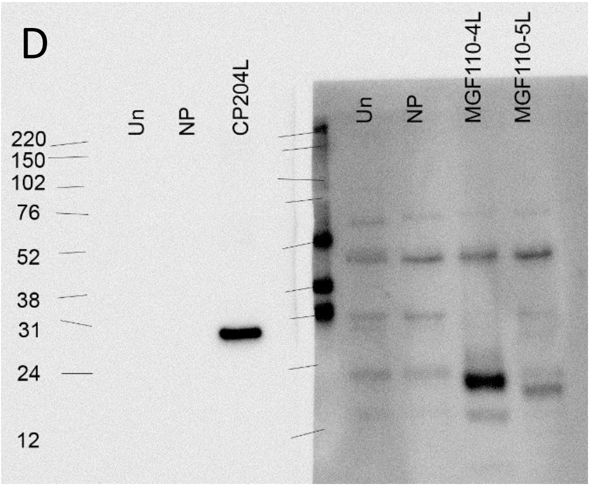

Supplemental Figure S4: MVA driven expression of ASFV genes. Cells were infected with MVA expressing the indicated ASFV genes or influenza NP and then either lysed (Panel A-D) or fixed (E) 48 hours (A) or 20 hours (B-E) later. Gene expression was determined by immunoblot or indirect immunofluorescence using anti-HA clone 3F10 (A-C, E), anti-ASFV p30 clone C18, or anti-XP124L R29 (D).

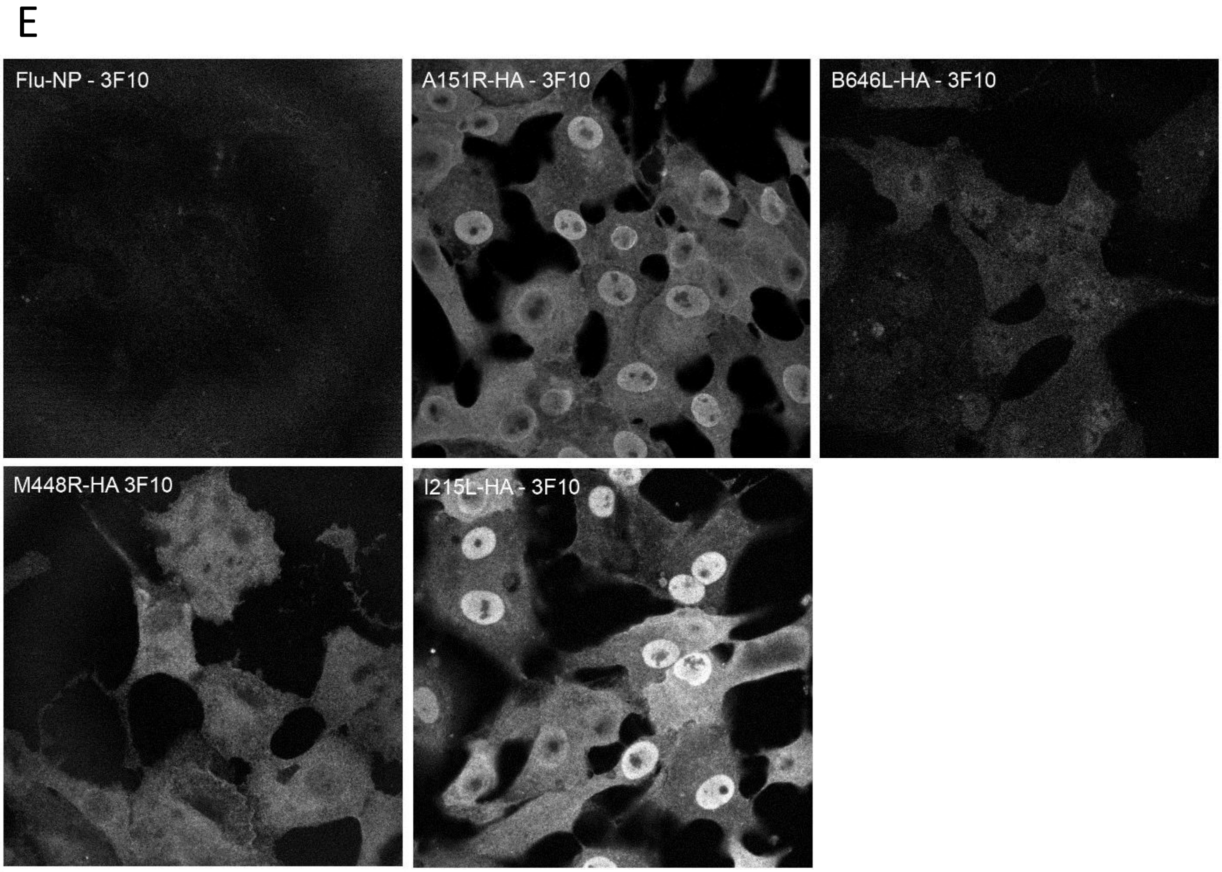

Supplementary References

1. Vydelingum S, Baylis SA, Bristow C, Smith GL, Dixon LK (1993) Duplicated genes within the variable right end of the genome of a pathogenic isolate of African swine fever virus. J Gen Virol 74: 2125-2130. doi: 10.1099/0022-1317-74-10-2125

2. Leitão A, Malur A, Cornelis P, Martins CLV (1998) Identification of a 25-aminoacid sequence from the major African swine fever virus structural protein VP72 recognised by porcine cytotoxic T lymphocytes using a lipoprotein based expression system. J Virol Met 75: 113-119. doi: 10.1099/0022-1317-82-3-513

3. Kollnberger SD, Gutierrez-Castaneda B, Foster-Cuevas M, Corteyn A, Parkhouse RM. Identification of the principal serological immunodeterminants of African swine fever virus by screening a virus cDNA library with antibody. J Gen Virol. 2002;83: 1331-1342. doi: 10.1099/0022-1317-83-6-1331.
